# Conformational states of the full-length glucagon receptor

**DOI:** 10.1038/ncomms8859

**Published:** 2015-07-31

**Authors:** Linlin Yang, Dehua Yang, Chris de Graaf, Arne Moeller, Graham M. West, Venkatasubramanian Dharmarajan, Chong Wang, Fai Y. Siu, Gaojie Song, Steffen Reedtz-Runge, Bruce D. Pascal, Beili Wu, Clinton S. Potter, Hu Zhou, Patrick R. Griffin, Bridget Carragher, Huaiyu Yang, Ming-Wei Wang, Raymond C. Stevens, Hualiang Jiang

**Affiliations:** 1 Drug Discovery and Design Center, State Key Laboratory of Drug Research, Shanghai Institute of Materia Medica, Chinese Academy of Sciences, 555 Zuchongzhi Road, Pudong, Shanghai 201203, China; 2 The National Center for Drug Screening and the CAS Key Laboratory of Receptor Research, Shanghai Institute of Materia Medica, Chinese Academy of Sciences, 189 Guo Shou Jing Road, Shanghai 201203, China; 3 Division of Medicinal Chemistry, Faculty of Sciences, Amsterdam Institute for Molecules, Medicines and Systems (AIMMS), VU University Amsterdam, De Boelelaan 1083, Amsterdam 1081 HV, The Netherlands; 4 The National Resource for Automated Molecular Microscopy, The Scripps Research Institute, 10550 N. Torrey Pines Road, La Jolla, California 92037, USA; 5 Department of Molecular Therapeutics, The Scripps Research Institute, 130 Scripps Way, Jupiter, Florida 33458, USA; 6 Department of Integrative Structural and Computational Biology, The Scripps Research Institute, 10550 N. Torrey Pines Road, La Jolla, California 92037, USA; 7 iHuman Institute, ShanghaiTech University, 99 Haike Road, Pudong, Shanghai 201203, China; 8 Department of Modeling and Structural Biology, Novo Nordisk, Novo Nordisk Park, Malov 2760, Denmark; 9 The CAS Key Laboratory of Receptor Research, Shanghai Institute of Materia Medica, Chinese Academy of Sciences, 555 Zuchongzhi Road, Shanghai 201203, China; 10 Departments of Biological Sciences and Chemistry, Bridge Institute, University of Southern California, 3430 S. Vermont Avenue, Los Angeles, California 90089, USA

## Abstract

Class B G protein-coupled receptors are composed of an extracellular domain (ECD) and a seven-transmembrane (7TM) domain, and their signalling is regulated by peptide hormones. Using a hybrid structural biology approach together with the ECD and 7TM domain crystal structures of the glucagon receptor (GCGR), we examine the relationship between full-length receptor conformation and peptide ligand binding. Molecular dynamics (MD) and disulfide crosslinking studies suggest that apo-GCGR can adopt both an open and closed conformation associated with extensive contacts between the ECD and 7TM domain. The electron microscopy (EM) map of the full-length GCGR shows how a monoclonal antibody stabilizes the ECD and 7TM domain in an elongated conformation. Hydrogen/deuterium exchange (HDX) studies and MD simulations indicate that an open conformation is also stabilized by peptide ligand binding. The combined studies reveal the open/closed states of GCGR and suggest that glucagon binds to GCGR by a conformational selection mechanism.

## Abstract (web summary)

G protein-coupled receptors (GPCRs) are the largest family of transmembrane signaling proteins in humans and exhibit diverse activation mechanisms. Here, the authors combine electron microscopy, hydrogen deuterium exchange and Molecular dynamics simulations to gain insight into the activation mechanism of the glucagon receptor.

## Graphical abstract

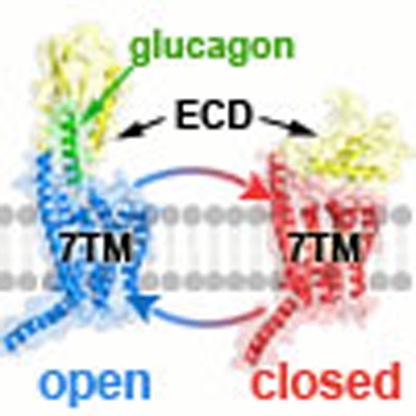

G protein-coupled receptors (GPCRs), the largest family of transmembrane signalling proteins in humans, can be divided into five main families/classes according to their sequence homology: Rhodopsin (class A), Secretin-like (class B1), Adhesion-like (class B2), Glutamate (class C) and Frizzled (class F)^1^. GPCRs share a common architecture of seven transmembrane helical (7TM) domains with a similar helical fold^2^,^3^,^4^,^5^,^6^ but structurally divergent loop regions and a structurally diverse N-terminal extracellular domain (ECD)^1^,^2^,^3^. Class A GPCRs recognize their endogenous ligands through an orthosteric site in the 7TM domain (or in the case of larger peptide ligands by a site formed as a combination of ECD and 7TM domain)^1^,^7^. Class C GPCRs recognize the endogenous small molecule ligands by orthosteric sites in the ECD^8^, while class F GPCRs bind the lipoprotein Wingless/Int-1 (WNT) in the ECD^9^. While adhesion-like class B GPCRs do not recognize ligands extracellularly^1^, secretin-like class B GPCRs bind their endogenous peptide ligands with both the ECD and 7TM domain^10^,^11^,^12^.

Class B GPCRs play causal roles in many diseases, ranging from diabetes and osteoporosis to anxiety. Pharmacological studies with truncated and chimeric peptide ligands^10^,^11^,^12^,^13^,^14^,^15^,^16^,^17^ together with ECD–ligand crystal structures^10^,^11^,^12^,^18^,^19^,^20^,^21^,^22^,^23^,^24^,^25^,^26^,^27^,^28^ provide the basis for a ‘two-domain' binding mechanism of peptide hormone ligands to secretin-like class B GPCRs in which: (i) the C terminus of the peptide ligand forms an initial complex with the ECD and this allows (ii) the N terminus of the peptide ligand to interact with the 7TM domain and to activate the class B GPCR^10^,^11^,^12^. Structures of ECDs of class B GPCRs alone or in complex with their peptide ligands have been determined using X-ray crystallography or NMR^10^,^11^,^12^,^18^,^19^,^20^,^21^,^22^,^23^,^24^,^25^,^26^,^27^,^28^, and have revealed information about ligand recognition and associated structural mechanisms^10^,^11^,^12^. Overall, the ECDs share a three-layer α-β-β/α architecture consisting of two pairs of antiparallel β-sheets (β1–β2 and β3–β4) and an N-terminal α-helix (αA), while the peptide ligands form a conserved α-helical segment in their C termini that binds to the ECD^10^,^11^,^12^. Recently, the crystal structures of the 7TM domains of two secretin-like class B members, the glucagon receptor (GCGR)^4^ and the corticotrophin-releasing factor-1 receptor^29^ were solved. Despite a similar arrangement of the transmembrane helices to previously determined GPCR structures, these two structures contain wider and deeper cavities in the ligand-binding pockets than class A GPCRs^4^,^11^. Moreover, based on the GCGR 7TM crystal structure (PDB: 4L6R), the GCGR ECD structure (PDB: 4ERS)^30^, the ECD structure of the GCGR homologue glucagon-like peptide-1 receptor (GLP-1R) bound to the GLP-1 (PDB: 3IOL)^19^ and the N-capped conformation of pituitary adenylate cyclase-activating polypeptide (PDB: 1GEA)^31^, a structural model of full-length glucagon-bound GCGR (now abbreviated as *glucagon*-GCGR) was constructed^4^. This model is consistent with the results of extensive mutation studies for GCGR^4^,^30^,^32^,^33^,^34^,^35^,^36^ and other class B GPCRs^11^, and offers a template for studying the structure–function relationship of the full-length GCGR.

The activation process of GPCRs involves a series of signalling states and each state is likely to be represented by an ensemble of conformations^37^. Current knowledge about the structure and function of class B GPCRs suggests that through binding with the ECD and the 7TM domain, the peptide ligand stabilizes conformational changes in the 7TM domain that facilitate receptor activation and signalling via G-proteins, arrestin or other factors^10^,^11^,^12^. Furthermore, peptide ligand may stabilize the active conformation of the 7TM domain and the orientation between the ECD and 7TM domain^10^,^11^,^12^. Previous chimera studies indicated that interactions between the ECD and the third extracellular loop (ECL3) of GCGR stabilize the inactive conformational state of GCGR, and that disruption of this ECD–ECL3 interaction in the GLP-1R-GCGR ECL3 chimera leads to increased basal receptor activity^30^. To understand the dynamics associated with the activation of class B GPCRs, investigations of the relative motions between the ECD and 7TM domain in a full-length receptor are required. For this purpose, we employ electron microscopy (EM), hydrogen/deuterium exchange (HDX), molecular dynamics (MD) simulations and site-specific disulfide bond crosslinking experiments to study the dynamic conformations of the ECD with respect to the 7TM domain in GCGR.

## Results

### EM map of antibody-bound full-length GCGR

**Figure 1:**
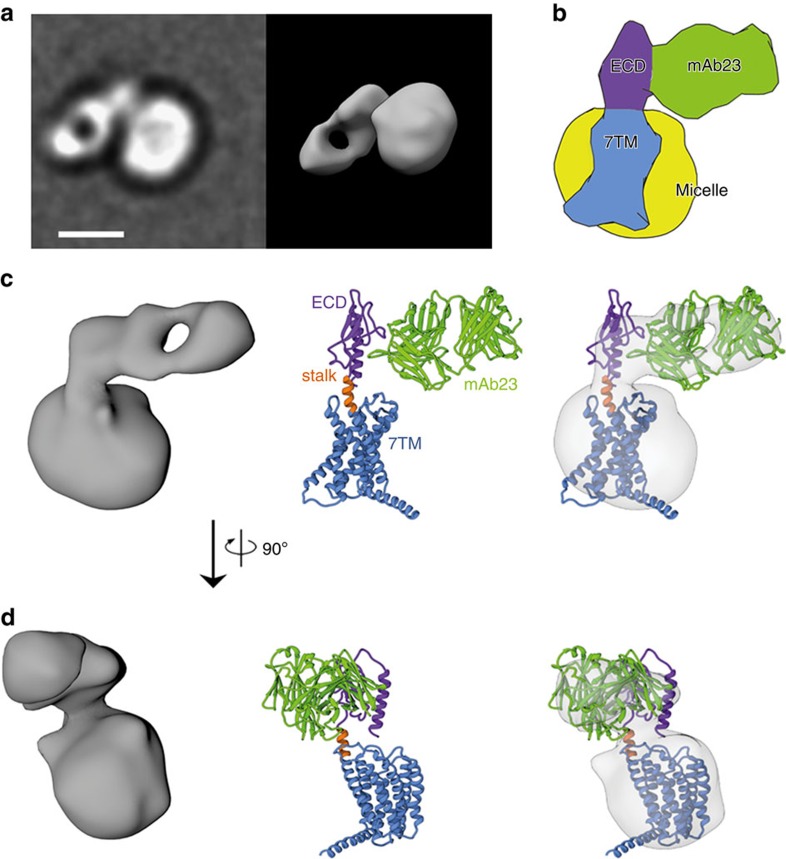
EM analysis of glucagon receptor–mAb23 complex. (**a**) Exemplary two-dimensional (2D) class average (sum of 343 individual particles) of negatively stained GCGR–mAb23 complex (left) and corresponding 3D surface representation of 3D map (∼30 Å resolution) determined using EM random conical tilt methods. 3D map is shown (right) in similar orientation to the 2D average (centre) and also rotated into an orientation convenient for comparison with the X-ray map. White scale bar, 50 nm. (**b**) Schematic interpretation of the domains in the EM map, rotated into an orientation convenient for comparison. (**c**) 3D envelope of the EM map (left), molecular model of the mAb23-bound full-length glucagon receptor structure based on mAb1-bound ECD (PDB code: 4ERS)^30^ and 7TM (PDB code: 4L6R)^4^ crystal structures (middle) and the molecular model fitted into the EM map (right). (**d**) View of panel c rotated 90° clockwise. Additional information on the EM maps is provided in Supplementary Fig. 1.

An EM map was determined for full-length GCGR in complex with the antigen-binding fragment (Fab) of the monoclonal antibody mAb23 (ref. 30; Fig. 1 and Supplementary Fig. 1). mAb23 shares a high sequence similarity with mAb1 (90% in light chain, 83% in heavy chain, Supplementary Fig. 2c) previously used to stabilize the crystal structure of the ECD region of GCGR^30^. This enabled us to derive a molecular model of the GCGR–mAb23 complex from the three-dimensional (3D) EM map that is consistent with the mAb1-bound GCGR ECD crystal structure and can explain similarities and differences in the ECD epitope maps of mAb1 and mAb23 (see Methods and Supplementary Fig. 2)^30^. The ensemble of EM maps clearly shows the central cleft between the light and heavy chains of the mAb23 Fab and indicates that mAb23 interacts with the ECD, but does not interact with the 7TM domain of GCGR (Fig. 1 and Supplementary Fig. 1). The EM map is in line with previous GCGR studies showing that mAb23 interacts with the ECD, preventing glucagon from binding to the receptor^30^, and shows a relative orientation of the ECD and the 7TM domain that is similar to the previously proposed hybrid glucagon-GCGR model^4^,^11^ based on separate 7TM (ref. 4) and mAb1-bound ECD^30^ crystal structures. The EM model suggests that mAb23 stabilizes GCGR in an open conformation in which the elongated transmembrane helix 1 (TM1) stalk region connects the ECD and 7TM domains and the ECD is almost perpendicular to the membrane surface (Figs 1c,d). It should be noted, however, that the EM map in principle allows slightly different orientations of the 7TM domain in the detergent micelle, and therefore alternative elongated orientations of the 7TM domain with respect to the ECD may be accommodated. Moreover, the ECD may adopt multiple conformations when the mAb23 antibody is not present, and other antibodies may bind different receptor conformations.

### Stabilization of the stalk region by peptide ligand binding

**Figure 2:**
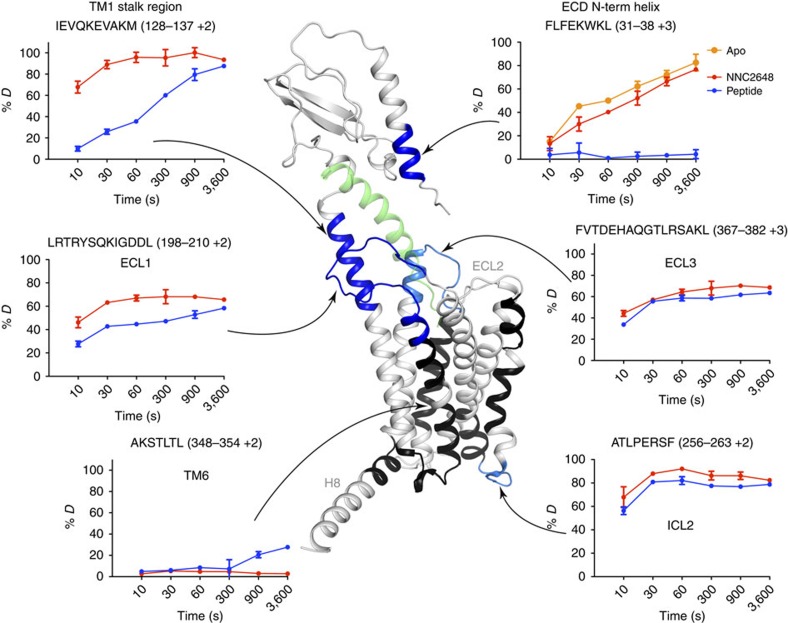
Stabilization of GCGR by peptide ligand in the HDX studies. Changes to average percent deuterium are shown on the full-length GCGR model based on ECD and 7TM crystal structures. Dark blue regions of receptor indicate areas of large decreased exchange in the presence of the des-His^1^-[Nle^9^-Ala^11^-Ala^16^]-glucagon-NH_2_ peptide ligand (depicted as green ribbon) and cyan indicates regions with smaller decreased exchange, while black indicates no significant change and white indicates regions where no peptide ions were detected using mass spectrometry. HDX plots for selected regions are shown around the structure. The data are shown as mean±s.d. of three independent experiments. Average percent deuterium values and percent deuterium values at 10 s are reported in Supplementary Fig. 4 and Table 1, respectively.

We carried out HDX experiments for apo-GCGR and GCGR bound to either small molecule (NNC2648)^38^ or peptidic des-His^1^-[Nle^9^-Ala^11^-Ala^16^]-glucagon-NH_2_ (ref. 39) antagonists (Fig. 2, Table 1 and Supplementary Figs 3 and 4). While HDX experiments for apo-GCGR obtained limited sequence coverage particularly for the apo state as compared with that obtained for other GPCRs^40^, experiments for GCGR bound to small molecule or peptide provide information on approximately half of the receptor. Reduced sequence coverage may be a result of stability or aggregation of this class B GPCR in the HDX buffers employed. Comparison of the observed peptide from the ECD region of apo-GCGR with that from the NNC2648-bound GCGR suggests that NNC2648 binds exclusively to the 7TM domain and does not affect the ECD (Fig. 2). HDX studies comparing small molecule and peptide antagonist-bound GCGR revealed differential HDX behaviour in peptides corresponding to the whole receptor. As shown in Fig. 2, three regions displayed increased protection (decreased exchange at 10 s) in peptide-bound GCGR compared with small molecule-bound GCGR: the N-terminal part of the ECD αA-helix (F31-L38), the TM1 stalk (I128-M137) and ECL1 (L198-L210). The protection of the ECD and ECL1 on peptide ligand binding is in agreement with the previously reported glucagon-GCGR model based on ECD and 7TM crystal structures and is supported by extensive mutation studies^4^,^33^,^34^. The peptide antagonist protects the stalk region, particularly at shorter exchange times (that is, decreasing the high deuterium exchange rate at 10 s from 60 to 7%, Table 1), indicating that the α-helical conformation in the GCGR 7TM crystal structure is stabilized by peptide ligand binding (Fig. 2)^4^. In the GCGR 7TM crystal structure, this TM1 helical stalk is stabilized by the BRIL fusion protein and helix 8 of the adjacent symmetric unit (Supplementary Fig. 5)^4^. No sequence coverage was obtained for ECL2 in our HDX experiments. It is possible that this region is naturally packed into the peptide-binding pocket even without ligand binding, preventing accessibility for peptic digest. Deuterium exchange of ECL3, which plays a role in glucagon binding^4^, is only decreased to a small extent in peptide ligand-bound- (36%) compared with NNC2648-bound GCGR (45%), indicating that the accessibility of this region does not significantly change on ligand binding. HDX studies demonstrate that intracellular loop 2 (ICL2) that is accessible in the GCGR 7TM crystal structure, but is not in the direct vicinity of the ligand-binding site, has high deuterium exchange rates (55–68%) with and without bound peptide ligand. The TM6 region has a consistently low deuterium exchange rate (3%), implying that this region maintains a stable α-helical structure that is not accessible in small molecule or peptide ligand-bound GCGR.

### Open and closed conformational states of full-length GCGR

**Figure 3:**
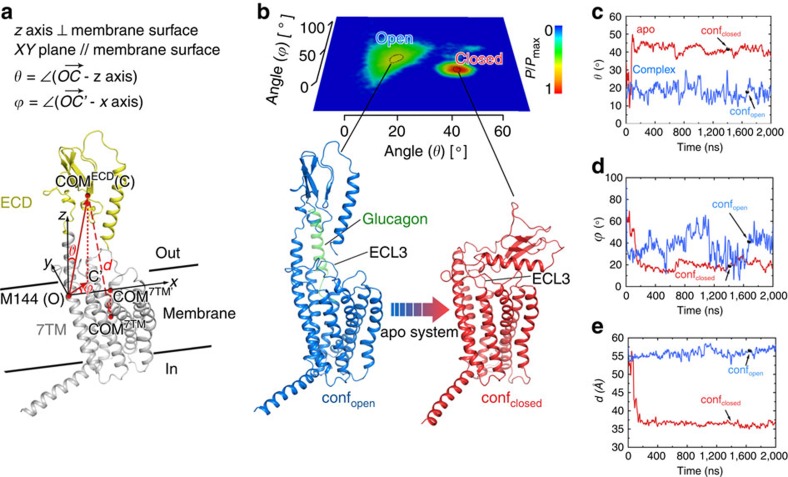
Motions of the ECD with respect to the 7TM domain in the simulations of glucagon-GCGR and apo-GCGR. (**a**) Definitions of the Cartesian coordinate system, the polar angle (*θ*, the included angle between vector **OC** and axis *z*), the Azimuthal angle (*φ*, the included angle between the **OC** projection on the *xy* plane and axis *x*) and the distance between the COMs of the ECD and 7TM domain (*d*) for describing the motions of the ECD with respect to the 7TM domain in the average structure in the simulation on the glucagon-GCGR complex. (**b**) The probability map of the MD snapshots with *θ* and *φ* as coordinates. The probability of the most abundant conformation is set to 1 and the relative probabilities of other conformations with respect to this conformation are shown. There are two states with higher probabilities (with *θ* and *φ* in areas circled by dotted lines): the open state (marine, represented by conf_open_) that can be stabilized by glucagon (semitransparent green cartoon) and the closed state (red, represented by conf_closed_). (**c**–**e**) Time dependences of *θ*, *φ* and *d* in the MD simulations on apo-GCGR and glucagon-GCGR and indication of conf_open_ and conf_closed_ MD snapshots.

To understand how the peptide ligand stabilizes the TM1 stalk and determines the relative orientation of the ECD and 7TM domains, we performed MD simulations of apo-GCGR and glucagon-GCGR embedded into a palmitoyloleoylphosphatidylcholine bilayer (Fig. 3a). The MD simulation of apo-GCGR revealed significant motions of the ECD that rotates and moves down towards the 7TM domain (Supplementary Fig. 6a and Supplementary Movie 1). Through structural superposition, we found that the whole TM1 and the stalk region bend around residue M144 in both the glucagon-GCGR and apo-GCGR MD simulations to facilitate motions of the ECD (Supplementary Fig. 6b). To give a clear description of the motions between the ECD and 7TM domains in the two systems, we constructed a Cartesian coordinate system by taking the Cα atom of M144 as its origin (designated as point O), the outward membrane normal as its *z* axis, the plane parallel to the membrane surface as the *xy* plane and the plane defined by the *z* axis and the centre of mass (COM) of the 7TM domain as the *xz* plane (Fig. 3a). In this Cartesian coordinate system, the polar angle *θ* and the Azimuthal angle *φ* of vector OC (linking the origin and the COM of ECD) can, respectively, describe the swing and rotation motions of the ECD in the simulations (Fig. 3a), and the distance *d* between the COMs of the ECD and 7TM domains represents one of the direct results of these motions (Fig. 3a). For apo-GCGR, the *θ* value increases from ∼20° to ∼50° during the first 150 ns and then fluctuates around 40° (Fig. 3c), indicating a large swing motion of the ECD towards the 7TM domain (Supplementary Movie 1). For the glucagon-GCGR complex, the *θ* value fluctuates around ∼20° (Fig. 3c), implying that the ECD undergoes a swing vibration around the point O (Supplementary Movie 2). The *φ* profile of glucagon-GCGR fluctuates significantly (Fig. 3d), suggesting that the ECD runs a rotation vibration around the *z* axis (Supplementary Movie 2). However, the relatively stable *φ* profile of apo-GCGR points to the stabilization of the ECD by the 7TM domain (Fig. 3d). Consequently, the distance between the ECD and 7TM domains of glucagon-GCGR is significantly larger than in apo-GCGR (Fig. 3e). A probability map with *θ* and *φ* as coordinates shows two clusters of conformations (Fig. 3b), representing ‘open' and ‘closed' states of the receptor. In the cluster (*θ*=15°∼25°, *φ*=20°∼40°) of the ‘open state' mainly revealed by the glucagon-GCGR simulation (represented by MD snapshot conf_open_), the ECD is stabilized by glucagon binding and is perpendicular to the membrane surface. In contrast, in the ‘closed state' cluster (*θ*=40°∼45°, *φ*=15°∼25°) observed in the apo-GCGR simulation (represented by MD snapshot conf_closed_) the extracellular surface of the 7TM domain is covered by the ECD. In this closed conformation the ECD interacts with the three ECLs (ECL1, ECL2 and ECL3) of GCGR (Fig. 3b). Particularly, the ECD has been implicated to negatively regulate GCGR through possible interactions with ECL3 (ref. 30).

### Peptide binding stabilizes the open conformation of GCGR

**Figure 4:**
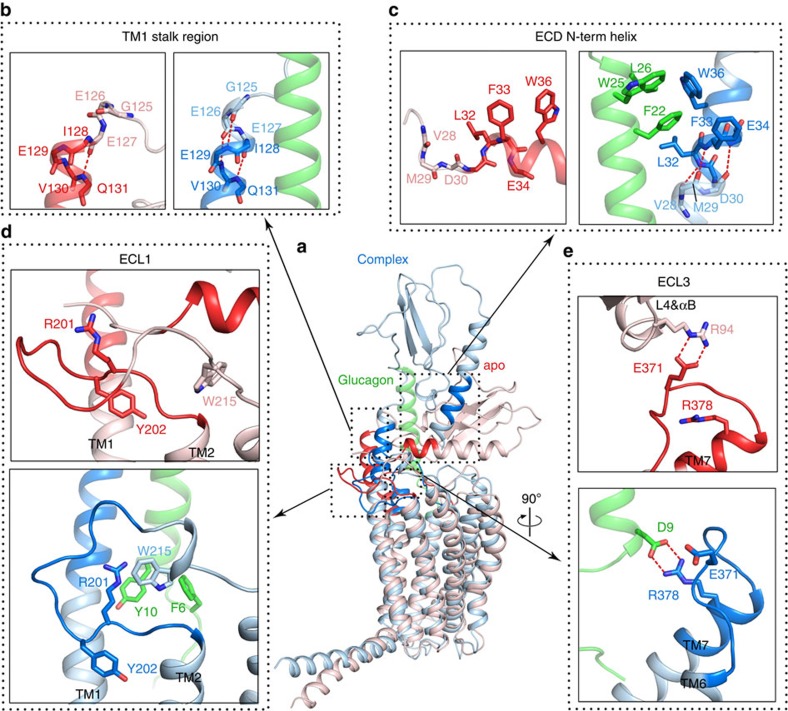
Comparison of open glucagon-bound and -closed apo-GCGR structures. (**a**) Representative snapshots of glucagon-bound GCGR (conf_open_, blue) and apo-GCGR (conf_closed_, red) in the MD simulations (defined in Fig. 3). Regions investigated in HDX studies (see Fig. 2 and Table 1) are coloured dark (red/blue) and shown in more detail in (**b**) the top region of the stalk; (**c**) the N terminus of the ECD; (**d**) ECL1; and (**e**) ECL3. Residues involved in specific interactions or adopting different conformations in glucagon-bound and apo-GCGRs are depicted as sticks.

The predicted accessibilities of amide protons based on MD simulations of apo-GCGR versus glucagon-GCGR correspond with the experimentally determined deuterium exchange rates at 10 s observed in HDX studies of NNC2648-bound- versus peptide antagonist-bound GCGR (Table 1). Both HDX studies and MD simulations consistently demonstrated that the ECD, TM1 stalk and ECL1 regions are stabilized by peptide ligand binding. The amide proton accessibility of the TM1 stalk (13%) in the simulation of apo-GCGR is still relatively low considering the high percentage of deuterium exchange (60%) of NNC2648-bound GCGR. The MD simulation nevertheless indicates that in the absence of a peptide ligand the top region of the stalk (G125 to I128) unwinds (Fig. 4b), which consequently increases the accessibility of the amide protons of following residues, for example, E129 and V130, in line with the HDX studies (Table 1). MD simulations suggest that peptide ligand binding stabilizes an extended conformation of αA in the ECD in which the V28–D30 fragment forms stable intrahelical hydrogen bonds with E34, F33 and L32 (Fig. 4c). An extended αA was also observed in ECD crystal structures of class B GPGRs^19^,^20^,^22^,^23^,^27^; however, it was not seen in GCGR crystallized with an antibody instead of its cognate peptide ligand (PDB: 4ERS)^30^. In line with the crystal structure, this fragment is dissociated from the αA-helix in the simulation of apo-GCGR (Fig. 4c). Similarly, glucagon binds a more compact fold of the ECL1 of GCGR that is stabilized by bulky residues R201 and Y202 and supported by interactions between bulky residues in glucagon (for example, F6/Y10) and GCGR (for example, W215). All the implicated residues in glucagon and GCGR have indeed been shown to play a role in glucagon binding^4^. Without glucagon, ECL1 in the apo-GCGR is exposed to the solvent environment and is very dynamic in the simulation (Fig. 4d and Supplementary Fig. 7), thereby resulting in an increased accessibility (Table 1). Notably, ECL3 could form stable salt bridges with glucagon (R378^ECL3^-D9^glucagon^, in line with previous mutation studies^4^) or with the L4 and αB of ECD (E371^ECL3^-R94^ECD^) in both simulations (Fig. 4e), which would stabilize the conformation of ECL3 and contribute to the stabilization of the closed state in apo-GCGR. Therefore, the accessibility of ECL3 changes little in the two systems (Table 1). As both ICL2 and TM6 are far away from the peptide-binding site, glucagon binding has few direct effects on their conformations and the proton accessibilities of ICL2 and TM6 do not alter in the two systems, an observation consistent with that seen in the HDX studies (Table 1).

### Transitions between open and closed conformations of GCGR

**Figure 5:**
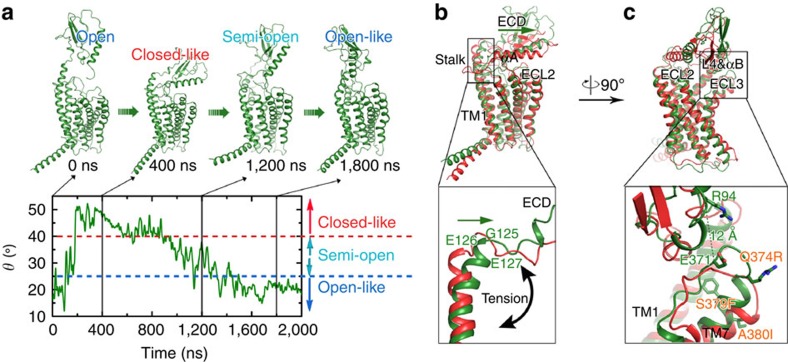
Conformational states of the ECL3 chimera in the MD simulation. (**a**) Orientations of the ECD with respect to the 7TM domain in the simulation on the ECL3 chimeric apo-GCGR. Time dependences of the polar angel (*θ*, defined in Fig. 3) in the simulation are shown at the bottom and typical snapshots taken from specific periods of the trajectories are displayed at the top. Comparison of conformations of the TM1 stalk region (**b**) and interaction between ECD and ECL3 (**c**) in the closed-like structure in the ECL3 chimera (green) with the closed state in the wild-type apo-GCGR (red). The snapshot at 300 ns in the simulation on the chimera and the structure of conf_closed_ (Fig. 3) are used. Residues with different conformations in the two structures are depicted as sticks and C_α_–C_α_ distance between R94 and E371 is labelled. For clarity, mutations are labelled in orange.

It has been previously demonstrated that the exchange of ECL3 in GCGR for ECL3 of GLP-1R, a change of only three amino acids (Q374R, S379F and A380I), leads to a significant increase in basal GCGR signalling^30^. To investigate the structural basis for this increased constitutive activity, we performed an MD simulation on this ECL3 chimera. The motions between the ECD and 7TM in the chimera could be described using the polar angle *θ* defined in Fig. 3. The chimeric apo-GCGR adopts a closed-like structure (*θ*>40°) at the beginning of the simulation (that is different from the closed structure of wild-type apo-GCGR) but then undergoes a transition to an open-like structure (*θ*<25°) and maintains so until the end of the simulation (Fig. 5a). In the closed-like structure adopted by the ECL3 chimera, the top region of the stalk (G125–I128) does not unwind as in the wild-type but bends towards the 7TM domain as a whole helix during the swinging motion of the ECD (Fig. 5b), affecting the relative orientation of the ECD with respect to the 7TM domain. In the closed-like state the ECD is further away from the centre of the helical bundle (Fig. 5b) and the N-terminal loop of the ECD forms dynamic contacts with ECL1 and ECL2, unlike the stable contacts between the αA of ECD and ECL2 in wild-type GCGR (Supplementary Fig. 8). The stabilization of the helical conformation of the stalk region may be the result of a stronger hydrophobic pattern between TM7 and the TM1 stalk contributed by the S379F mutation (Supplementary Fig. 8). The tighter hydrophobic packing of TM7 and TM1 in the ECL3 chimera results in the loss of the stable salt bridge between R94^ECD^ and E371^ECL3^ (Fig. 5c) that is observed in wild-type GCGR (Fig. 4e). Except for S379F, the other two mutations do not seem to contribute to the conformational transition of the chimera.

**Figure 6:**
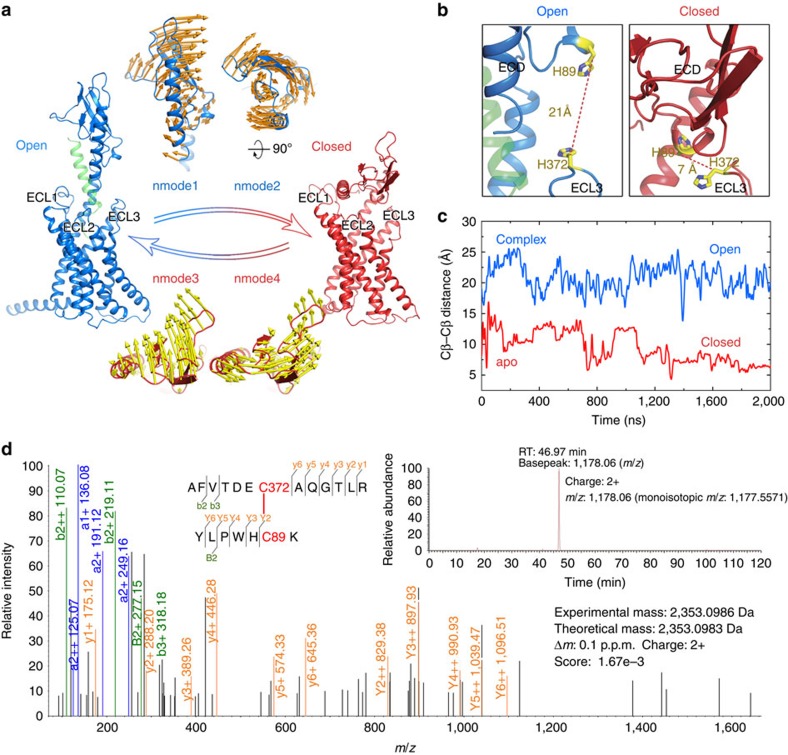
Transition between the two states and intervention by disulfide crosslinking studies. (**a**) Modes 1 and 2 of the NMA on the open structure (top, orange arrow), and modes 3 and 4 of the NMA on the closed structure (bottom, yellow arrow) based on representative open state (conf_open_) and closed state (conf_closed_) structures (Fig. 3). The vectors representing both the amplitudes and directions of residues during the conformational changes are mapped on the ECD. (**b**) Selected residue pair H372^ECL3^–H89^ECD^ for cysteine substitution is shown in the closed state of apo-GCGR, with the average Cβ–Cβ distance in the last 1,000 ns simulation. (**c**) Time dependences of the Cβ–Cβ distance between H372^ECL3^ and H89^ECD^ in the MD simulations on glucagon-GCGR (blue) and apo-GCGR (red). (**d**) MS/MS spectra of the HCD fragmentation of the doubly charged disulfide-containing peptide are shown; *b*, *y, B* and *Y* indicate types of fragment ions. Graphical fragment map correlates the fragmentation ions to the peptide sequence in which the disulfide-linked cysteine residues C89 and C372 are shown in red. The top-right panel shows a LC-MS analysis-extracted ion chromatogram of GCGR from *Spodoptera frugiperda (Sf*9*)* cells with chymotrypsin and trypsin digestion, representing the doubly charged crosslinked peptide between YLPWHC(89)K and AFVTDEC(372)AQGTLR through a disulfide bond.

Normal mode analysis (NMA)^41^ is an efficient method for predicting inherent flexibilities in biological macromolecules. We performed NMA on the typical structures of the open and closed states, that is, conf_open_ and conf_closed_ in Fig. 3b, to detect the intrinsic motions of GCGR. The low-frequency modes of GCGR produced by the NMA reflect the global motions of the receptor and are often related to biological functions^42^. The first two lowest-frequency motion modes (modes 1 and 2) on the open state are relevant to the transition from open to closed: in mode 1, the ECD moves downwards to the 7TM domain; in mode 2, the ECD undergoes an intrinsic rotation (Fig. 6a). Interestingly, NMA of the closed state (conf_closed_) revealed that the closed state has an intrinsic potential to change back to the open conformation (Fig. 6a). However, the modes relevant to this transition are only among the third and fourth lowest-frequency motion modes. Therefore, unless an external force or ligand is present, GCGR would favour the closed state within the circuit. The consistency between the results of MD and NMA supports the efficiency of both methods in studying the large-scale motions between the ECD and 7TM domains.

### Locking GCGR in its closed state by disulfide crosslinking

**Figure 7:**
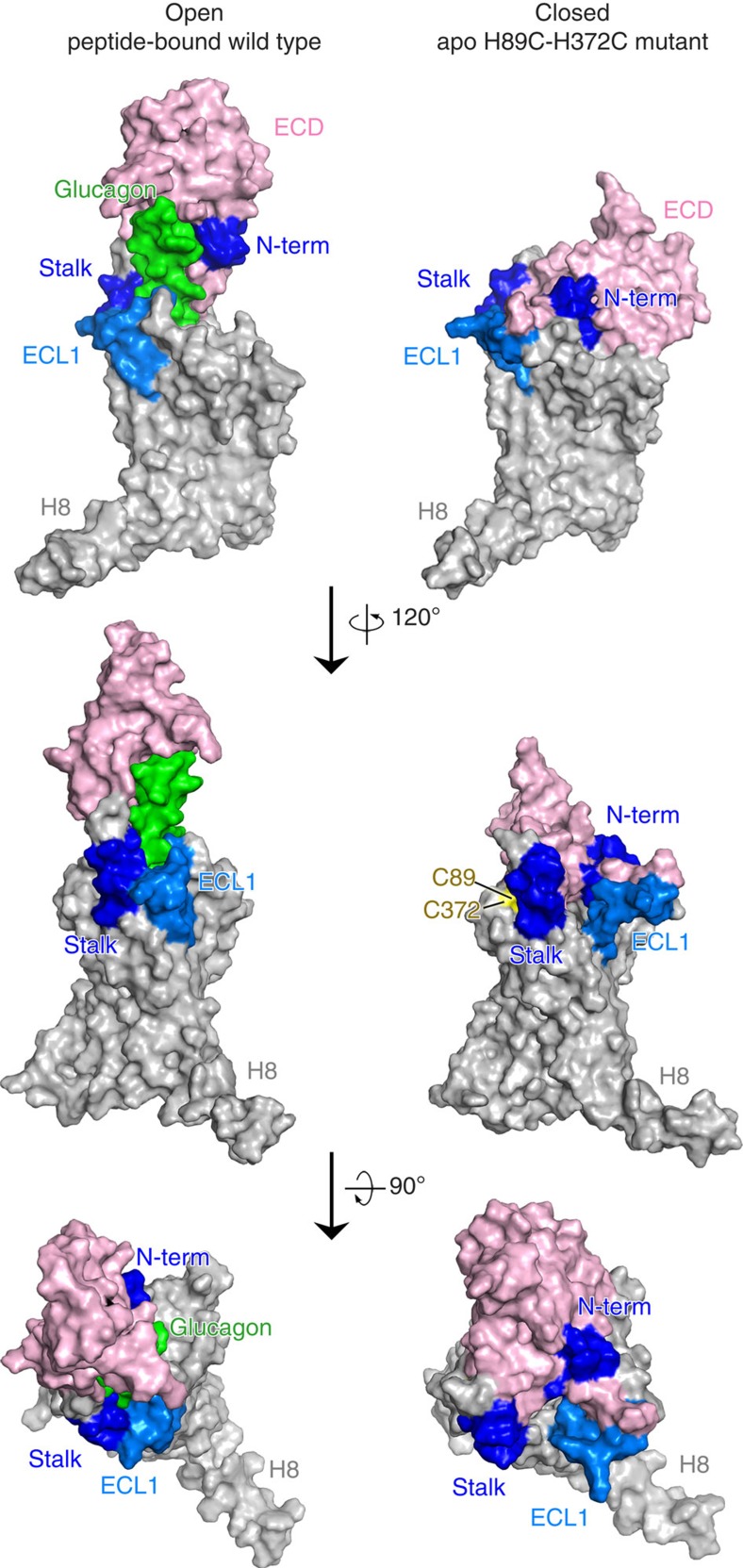
HDX studies of open peptide ligand-bound wild-type GCGR versus closed apo H89C/H372C mutant GCGR. Changes to average percent deuterium of extracellular regions observed in HDX studies are shown on full-length models of peptide ligand-bound wild-type GCGR (left) and closed H89C/H372C mutant GCGR (right). The N-terminal region of the ECD (N-term) and TM1 stalk are depicted in dark blue, indicating large decreased exchange in the presence of the des-His^1^-[Nle^9^-Ala^11^-Ala^16^]-glucagon-NH_2_ peptide ligand (green), while the smaller decreased exchange of the ECL1 region is coloured cyan. The ECD is coloured magenta, while residues C89 and C372 are coloured yellow. Differences in average percent deuterium values are reported in Supplementary Table 2.

In the closed state, all three of the ECLs (ECL1, ECL2 and ECL3) of the 7TM domain can interact with the ECD. To further validate the ECD–7TM interface in the closed state, we performed disulfide crosslinking studies to lock the closed conformation of GCGR. As residues in ECL1 and ECL2 loops play an important role in glucagon binding^4^, we focused on the design of a disulfide crosslink between the ECD and ECL3. In the open state, the ECD is far away from ECL3, while they are in close proximity to each other in the closed state (Fig. 6a), in which the average Cβ–Cβ distance of H89^ECD^–H372^ECL3^ in the last 1,000-ns trajectories of the apo-GCGR is only 7 Å (Figs 6b,c). Our experiment with the H89C/H372C GCGR mutant showed a significant decrease in glucagon-binding affinity (40% of the wild-type), which was rescued by adding 1 mM dithiothreitol (DTT; 114% of the wild-type; Supplementary Fig. 9 and Supplementary Table 1). The same reducing agent did not influence the binding potency of the H89C and H372C single-site mutants (Supplementary Fig. 9). This result suggests the formation of a disulfide bond between C89C and C372 in the majority of the receptor population, which otherwise would not exist in the open conformation because the Cβ–Cβ distance of H89^ECD^–H372^ECL3^ is much larger (Fig. 6c and Supplementary Fig. 10). The C89^ECD^–C372^ECL3^ disulfide bond in the H89C/H372C GCGR mutant was further validated in liquid chromatography–tandem mass spectrometry (LC-MS/MS) experiments. In the full-scan mass spectrum of the H89C/H372C GCGR mutant sample with chymotrypsin and trypsin digestion, evident triply (*m*/*z* 785.3722) and doubly (*m*/*z* 1,177.5571) charged peaks corresponding to the C89–C372 disulfide-containing peptide (theoretical molecular mass, 2,353.0983 Da) were observed (Fig. 6d and Supplementary Fig. 10b). Further higher-energy collisional dissociation (HCD) fragmentation of the YLPWHC(89)K-AFVTDEC(372)AQGTLR peptide generated a variety of crosslinked fragment ions, including Y2, Y3, Y4 and Y5 at YLPWHCK peptide side, indicating the presence of a disulfide bond between C89 and C372 (Fig. 6d and Supplementary Fig. 10d). The mass spectrometry results provide strong evidence that a disulfide bond was formed between C89 and C372 in the H89C/H372C GCGR mutant. While HDX experiments for wild-type apo-GCGR yielded only a limited sequence coverage for the ECD region (Fig. 2, Table 1 and Supplementary Fig. 4), percent deuterium exchange values could be obtained for the H89C/H372C mutant apo-GCGR for ECD, TM1 stalk, ECL1, ICL1, ICL2 and C-terminal regions (Fig. 7 and Supplementary Table 2). This allowed the identification of extracellular ECD, TM1 stalk and ECL1 regions in GCGR that are protected by peptide ligand binding compared with wild-type NNC2648-bound GCGR (Fig. 2) as well as H89C/H372C mutant apo-GCGR (Fig. 7). These three regions are indeed lining the peptide ligand-binding site and are solvent-exposed in apo-GCGR in full-length GCGR structural models. Hence, the combined radioligand binding, LC-MS/MS and HDX crosslinking studies suggest that there exists a conformational transition of the full-length GCGR on the cell surface that is in agreement with our MD simulation studies.

## Discussion

The EM structure of a full-length class B GPCR presented in this study supports an ‘open' conformational state, in which the ECD is almost perpendicular to the membrane surface. In this mAb23 antibody-stabilized open state, the ECD is connected to the 7TM domain via the TM1 stalk^4^ region (Fig. 1). HDX studies in combination with microsecond MD simulations indicate that an open receptor conformation is also stabilized by peptide ligand binding, although it must be emphasized that this open conformation could be different from the mAb23-bound conformation observed in the EM studies. In the absence of a peptide ligand, GCGR can also adopt a closed conformation, in which the ECD covers the extracellular surface of the 7TM domain (Figs 2 and 3 and Table 1). The HDX studies are consistent with the previous crystal structures of class B GPCRs^4^,^29^. In the GCGR crystal structure, ICL2 (residues L255–F264) is very dynamic with an average temperature factor above 180 (ref. 4). This is in agreement with the HDX studies in which we identified a similar fragment (A256–F263) showing high deuterium exchange (Table 1). Our HDX studies indicate that ECL1 is protected on peptide ligand binding (Figs 2 and 7). This suggests that the peptide is able to make unique interactions with this region of the receptor and possibly stabilize ECL1, which is unstructured in the GCGR crystal structure^4^. While the extended TM1 helix observed in the X-ray structure and EM map seems to be stabilized by lattice packing and antibody binding, respectively, the HDX studies provide complementary insights into the structural dynamics of the TM1 stalk. Our MD simulations match these HDX data and demonstrate how flexibility of the stalk region facilitates the transition of GCGR between closed and open states.

This is the first report of the putative closed state of GCGR identified by MD simulations, consistent with HDX results and validated by disulfide crosslinking studies (Figs 3, 4, 5, 6). The open and closed state model explains the differences in HDX results for peptide bound versus unbound GCGR (Figs 2 and 7). MD and NMA provide insights into the transition mechanism between these two conformational states. The NMA shows that apo-GCGR can easily adopt the closed state through the lowest-frequency motion modes; importantly, it also has the potential to return to the open state (Fig. 6). Such a large conformational change of GCGR would need a tensile force exerted by an immobilized ligand^43^, but not a flexible ligand such as glucagon^14^,^44^, which makes a ligand-induced fit mechanism less plausible. The transition between GCGR conformations is therefore proposed to occur via a conformational selection mechanism in which GCGR equilibrates between open and closed states. The closed state in which the ECD tightly contacts with the 7TM domain is energetically more favourable in the unbound receptor, while a peptide ligand preferentially binds the open conformation that allows the ligand to dock both the N-terminal loop and C-terminal helix to the 7TM domain and ECD, respectively. Our MD simulations were successfully used to design disulfide crosslinks between the ECD and ECL3 that fix GCGR in the closed state (Figs 6 and 7). Previous mutation studies showed that interactions between the ECD and ECL3 stabilized the inactive state of GCGR^30^, suggesting that there may be a link between open versus closed receptor conformations and activation states. The MD simulations show that the constitutively active GCGR-GLP-1R ECL3 chimera^30^ was unable to maintain a stable closed conformation and this adopted an open-like structure. Although the mutated residues in the ECL3 chimera are not directly involved in the interactions with the ECD in wild-type apo-GCGR, the S379F mutation strengthens the hydrophobic interactions between TM7 and the TM1 stalk. The stabilization of the TM1 stalk consequently affects the relative orientation of the ECD with respect to the 7TM domain in an open-like conformation (Fig. 5).

The proposed transition mechanism between open and closed conformational states is consistent with the ‘two-domain' ligand-binding model in which the C-terminal region of the ligand interacts with the ECD of class B GPCRs, and this facilitates the N-terminal region of the peptide ligand to interact with the 7TM domain and activate the receptor^10^,^11^,^12^. This two-step ligand recognition mechanism is supported by ECD–ligand crystal structures and pharmacological studies with truncated receptors and peptide ligands. N-terminally truncated forms of the CRF_1_, glucose-dependent insulinotropic peptide (GIP), GLP-1, glucagon and parathyroid hormone (PTH) peptides are competitive antagonists that display only a small decrease in the affinity for their corresponding receptor, while C-terminally truncated ligands remain active but bind the receptor with significantly decreased affinity^13^,^14^,^15^,^16^,^17^. Pharmacological and structural studies show that isolated ECDs of GLP-1, PTH_1_, CRF_1_ and CRF_2_ are still capable of binding peptide ligands^10^,^16^,^18^,^19^,^20^,^23^,^26^,^27^. Our studies show that peptide ligand interactions with the ECD, ECL1 and, to a lesser degree, with ECL3 stabilize the open state of GCGR and demonstrate the role of conformational flexibility in the GCGR ligand-binding process.

The existence of several conserved structural features and ligand interaction hotspots in secretin-like class B GPCRs suggests that the proposed flexible GCGR-ligand-binding model can partially be translated to other receptors. First of all, the crystal structures of the ECD–peptide complexes of different class B GPCRs show a conserved binding mode of the C-terminal α-helix of the peptide ligand between the two β-sheets of the ECD^12^. Second, mutagenesis and photo-crosslinking studies have identified several common interaction hotspots in the 7TM domain for binding the N-terminal region of the peptide ligand^11^. The full GCGR model as well as the recently reported CRF_1_ model^4^,^45^, based on ECD and 7TM crystal structures, can account for these experimentally supported interactions of the N-terminal region of peptide ligands with ECLs and residues located deep in the helical bundle^4^,^32^,^33^,^34^,^35^,^36^,^45^,^46^,^47^. Third (combined ligand and receptor), mutation and crosslinking studies suggest that the six homologous N-terminal residues of glucagon^32^,^36^, GLP^16^,^48^, GIP^49^, secretin^50^ and vasoactive intestinal peptide^51^ adopt similar binding modes in the 7TM of their respective class B GPCRs^11^. Differences in specific residues in the 7TM helical bundle and in the composition and length of ECLs of glucagon, GLP-1, GLP-2, GIP, secretin, VPAC_1_ and VPAC_2_ receptors may nevertheless result in different receptor–ligand interactions and structural dynamics. ECL1 for example, which is stabilized by peptide ligand binding in GCGR (Figs 2, 3 and 7) and plays a role in ligand binding in GCGR, GLP and other secretin receptors^33^,^34^,^52^,^53^, has a variable loop length among secretin-like class B GPCRs (15–41 residues), which is expected to affect loop flexibility and receptor–ligand interactions. Furthermore, the TM1 stalk helix may be shorter or less stable in other class B GPCRs compared with the long extended TM1 helix, consistent with EM (Fig. 1) and HDX (Figs 2 and 7) studies, and observed in the GCGR crystal structure^4^ and glucagon-GCGR MD simulations (Figs 3 and 4). Differences in the length and composition of peptides will determine their binding mode and flexibility as well. The N-terminal regions of peptide ligands that bind CRF_1_ and CRF_2_ are significantly longer than in the peptide ligands of other class B GPCRs^10^,^11^. Recent crosslinking studies, for example, indicate that the thirteen terminal residues of Urocortin-I (Ucn 1) adopt a somewhat different binding mode in CRF_1_ than the six N-terminal residues of glucagon in GCGR, although both receptor–ligand complexes share several common interaction sites in the TM7 domain^4^,^11^,^14^,^30^,^32^,^36^,^45^,^47^,^54^. Finally, it should also be noted that differences in the interactions between the ECD and the ligand C terminus may affect the binding mode (flexibility) of the ligand N terminus with the TM7 domain, as suggested by, for example, comparisons of GLP- and Ex-4-bound GLP-1R crystal structures and mutation studies^16^,^19^,^22^. Altogether, this indicates that the dynamic GCGR-glucagon binding model provides a useful template to guide the design of new experiments to investigate class B GPCR structure–function relationships.

The relative movement and interaction dynamics of the structured ECD and 7TM domains via the TM1 stalk pivot point may be an exclusive feature of class B GPCRs. The conserved structure of the ECD domain of class B GPCRs (100–160 residues^12^) is very different from the conserved ECD structure of class C GPCRs (500–600 residues^8^), class F GPCRs (200–300 residues^6^) and the structurally diverse N-terminal regions of class A GPCRs (4–80 residues^2^). Class C GPCRs act as dimers and conformational changes between the agonist-binding pockets in the extracellular Venus Fly Trap domains and the 7TM domains are mediated by a rigid and structured cysteine-rich domain^1^,^8^. This makes large movements of the Venus Fly Trap domain and interactions with the 7TM domain, similar to those between the ECD and 7TM domains of class B GPCRs, unlikely. The ECD linker domain of class F GPCRs is less rigid and may allow the WNT protein-binding cysteine-rich domain to move towards the 7TM domain. Most class A GPCRs have a relatively short N terminus; however, in some protein-binding class A GPCR subfamilies, such as chemokine receptors, the ECD plays an important role in endogenous ligand binding^55^. Similar to class B GPCRs, chemokine receptors bind their ligands via a two-step binding mechanism in which the structured C-terminal region of the chemokine binds the N-terminal region and ECLs of the receptor, and this allows the unstructured N terminus of the chemokine to target the 7TM helical bundle^55^,^56^,^57^. Efforts to obtain static structural information of full-length non-class A receptors are ongoing in many laboratories and the results will certainly be insightful. To date, however, it has been very challenging to obtain a full-length non-class A GPCR structure at atomic resolution, probably because of the dynamic nature and lack of ligands that exist to stabilize the different domains at the same time. Critical to fully understanding how these receptors work are the hybrid methods described here and correlating GPCR structure to function.

## Methods

### EM studies

Full-length GCGR was purified as follows^4^. *Sf*9 membranes were prepared with one wash cycle of hypotonic buffer (25 mM HEPES, pH 7.5, 10 mM MgCl_2_ and 20 mM KCl) in the presence of EDTA-free protease inhibitor cocktail tablets (Roche) and four wash cycles of high-salt buffer (25 mM HEPES, pH 7.5, 1 M NaCl, 10 mM MgCl_2_ and 20 mM KCl). Two grams of washed membranes containing the full-length construct were resuspended in 30 ml of buffer (25 mM HEPES, pH 7.0, 166 mM NaCl and 13.3% glycerol) and incubated with 270 μM of compound NNC0640 for 30 min at room temperature. The receptor was solubilized with 1/0.1% (w/v) of n-dodecyl-β-D-maltopyranoside (Anatrace) and cholesteryl hemisuccinate (Sigma; DDM/CHS) for 2 h at 4 °C. The insoluble material was pelleted by ultracentrifugation in a Ti70 rotor at 504,300 *g* for 30 min at 4 °C. The NaCl and DDM/CHS concentrations of the supernatant were adjusted to 800 mM and 0.5/0.05%, respectively, by adding equal volume of talon-binding buffer (25 mM HEPES, pH 7.0, 1.475 M NaCl and 10% glycerol). Protein was bound to 2 ml of talon superflow resin slurry (Clontech) overnight at 4 °C on a rotator in the presence of 15 mM imidazole, pH 7.5, and 100 μM NNC0640. The talon resin was washed with 10 × bed volume of wash buffer 1 (25 mM HEPES, pH 7.0, 800 mM NaCl, 10% glycerol, 0.04/0.008% DDM/CHS, 30 μM NNC0640, 40 mM imidazole, pH 7.5). Detergent concentration was lowered by washing the resin with 20 × bed volume of wash buffer 2 (25 mM HEPES, pH 7.0, 500 mM NaCl, 10% glycerol, 0.02/0.004% DDM/CHS and 30 μM NNC0640). The protein was eluted with 2.5 ml of elution buffer (25 mM HEPES, pH 7.0, 150 mM NaCl, 10% glycerol, 0.02/0.004% DDM/CHS, 30 μM NNC0640, 300 mM imidazole, pH 7.5). After purification in a DDM/CHS-based detergent system, the samples are incubated with 1% lauryl maltose neopentyl glycol to exchange the detergent. The mAb23 Fab (kindly provided by Genentech) was mixed with the receptor sample followed by size exclusion chromatography to isolate the complex. EM samples were prepared as follows^58^. Briefly, protein solution was applied on freshly glow-discharged carbon-coated copper grids and negatively stained three times using 2% uranyl formate. Images were acquired with a Tecnai F20 Twin transmission electron microscope operating at 200 keV, an electron dose of ∼45 e^−^ Å^−2^ and nominal underfocus of 0.7–1.7 μm. In total, 648 tilt-pair images (0° and −50°) were automatically collected at a nominal magnification of × 62,000 (representing a pixel size of 0.273 nm) on a Tietz F415 4 K × 4 K charge-coupled device camera using the Leginon data collection software^59^. EM samples were diluted using the SEC flow through buffer just before grid preparation. Dilutions were optimized to ensure a good distribution of the particles across the grid substrate (neither too crowded nor too sparse), and the final concentration of protein sample used was typically ∼0.01 μM. Experimental data were processed with the Appion software package^60^ interfaced with the Leginon database infrastructure. A total of 49,531 tilt-pair particles were automatically selected using a difference of Gaussian algorithm^61^ and extracted with a box size of 80 pixels. Class averages were calculated using the XMIPP reference-free maximum likelihood alignment algorithm^62^. Class averages were manually inspected, and class averages that did not represent a meaningful structure were identified and particles belonging to these class averages were removed from the particle stack. This process was repeated twice. The remaining particles were used as references for Spider two-dimensional alignment followed by Coran classification^63^. All 165 classes that were produced using this procedure were used to calculate 3D maps on the basis of the matching tilt-pair particles and using random conical tilt geometry^64^. The maps were then divided into three groups: Group 1 (representing 33% of the particles) includes the volumes presented in Fig. 1 and the entire set shown in Supplementary Fig. 1, and shows the GCGR–mAb23 Fab complex in one preferred orientation. Group 2 (representing 20% of the particles) shows the complex in an alternative preferred orientation (Supplementary Fig. 1). In both Groups 1 and 2 the maps indicate that the mAb23 Fab fragment is bound in a very similar position in relation to full-length GCGR. The central cleft between the heavy and light chains of the mAb23 Fab, which is clearly visible in the EM maps of Group 1, provides a validation of the quality of the 3D volume (Supplementary Fig. 1 and Fig. 1). We therefore used maps from Group 1 for further data analysis and 3D EM model construction. The remaining 47% of the particles resulted in maps that did not yield a clearly interpretable volume; this percentage of discarded particles maps is, in our experience, a typical outcome for random-canonical tilt reconstructions and we assume results from a combination of badly picked particles, damaged particles or artefacts of sample preparation (uneven or thin stain, and so on). The volume shown in Fig. 1 contains 343 particles and has a resolution of 34 Å (FSC 0.5 criterion).

### Modelling mAb23-bound GCGR in EM map

A 3D model of the mAb23 Fab was constructed with Molecular Operating Environment (Chemical Computing Group Inc.) on the basis of the crystal structure of the mAb1 Fab (PDB code: 4ERS)^30^. The amino-acid sequences of mAb23 (WO 2013/059531 A1) and mAb1 (ref. 30) Fab fragments share high sequence similarity (90% in light chain and 83% in heavy chain, Supplementary Fig. 2c). Although the EM map is not of sufficient resolution to provide atomistic information on mAb23–ECD interactions, the mAb23-bound GCGR model derived from the EM map is in line with similarities and differences between mAb1 and mAb23 epitopes. The mAb1-bound GCGR ECD crystal structure and mAb23–GCGR EM model indicate that overlapping mAb1/mAb23 epitopes Y65, L85 and W87 are in the vicinity of the H3 loops of mAb23 (W320) and mAb1 (L320; Supplementary Fig. 2a,b), while K90 and R94 stabilize the position of L85 and W87 by interacting with the stalk/region connecting the ECD and the 7TM domain of GCGR. The mAb1–ECD crystal structure^30^ further shows that the mAb1-specific epitope Y84 interacts with the mAb1-specific I319 residue located in the H3 loop of mAb1 that is three residues shorter in mAb23 (Supplementary Fig. 2c). Other mAb1-specific epitopes may stabilize the position of Y84 by direct aromatic stacking (Y39 and W83) or by forming hydrogen-bond interactions at the turn between β-strands 2 and 3 (T57). The mAb23–GCGR EM model suggests that the mAb23-specific epitope F62 is in the vicinity of the mAb23-specific Y269 residue (S268 in mAb1), while the mAb23-specific epitope L50 may stabilize the position of F62 by hydrophobic interactions at the interface of α-helix 1 and β-strands 1–2 (Supplementary Figs 2a,b).

### HDX studies

HDX of GCGR without the T4–lysozyme insert was carried out at 4 °C as follows. Briefly, the receptor was incubated in a D_2_O buffer for a range of exchange times from 10 s to 1 h before quenching the deuterium exchange reaction with an acidic quench solution (pH 2.4). All mixing and digestions were carried out on a LEAP Technologies Twin HTS PAL liquid-handling robot housed inside a temperature-controlled cabinet^40^. Digestion was performed in line with chromatography using an immobilized pepsin column. Mass spectra were acquired on an linear ion trap (LTQ) Orbitrap XL ETD mass spectrometer and percent deuterium exchange values for peptide isotopic envelopes at each time point were calculated and processed using the HDX Workbench software^65^.

The following quality criteria were used: (1) HDX data were considered only under the following conditions: i) The data contained a validated peptide set for which the monoisotopic mass had less than a 3-p.p.m. mass error, ii) the fragmentation spectrum when evaluated by Mascott (Matrix Science, UK) had an ion score of no less than 20, iii) the ion score exceeded the false discovery rate as determined using a decoy database, and iv) the cleavage sites did not violate the preference for pepsin and were manually confirmed; (2) for the HDX data to be included in the perturbation table, the peptide must be detected in all of the exchange time points and all of the three replicates (42 injections per sample). The differential analysis only includes data for peptides detected in all 84 injections (42 injections per comparison). There are over 1,368 individual %*D* values in the data set presented (triplicate values at six time points for 76 peptides). Alternative methods to sample preparation are now emerging for HDX that may be beneficial depending on the specific sample^66^; however, for the sake of consistency within different techniques, particularly the crystallographic data, we have selected the detergent-solubilized state of the receptor.

### Construction of GCGR mutants and cell transfection

The complementary DNA encoding the human GCGR was originally obtained from GeneCopoeia and cloned into the expression vector pcDNA3.1/V5-His-TOPO (Invitrogen) at the HindIII and EcoRI sites. The single and double mutants were constructed using PCR-based site-directed mutagenesis. CHO-K1 cells were seeded on 96-well poly-D-lysine-treated cell culture plates (PerkinElmer) at a density of 3 × 10^4^ cells per well. After overnight culture, the cells were transiently transfected with wild-type or mutant GCGR DNA using Lipofectamine 2000 transfection reagent (Invitrogen).

### Whole-cell glucagon-binding assay

Cells were harvested 24 h after transfection, washed twice and incubated with blocking buffer (F12 supplemented with 33 mM HEPES, pH 7.4 and 0.1% BSA) for 2 h at 37 °C. Cells were treated with PBS or DTT for 10 min before homogeneous binding. They were then washed twice with PBS and were incubated in binding buffer with constant concentration of ^125^I-glucagon (40 pM) and different concentrations of unlabelled glucagon (3.57 pM∼1 μM) at room temperature for 3 h. Cells were washed three times with ice-cold PBS and lysed by 50 μl lysis buffer (PBS supplemented with 20 mM Tris-HCl, 1% Triton X-100, pH 7.4). The plates were subsequently counted for radioactivity (counts min^−1^) in a scintillation counter (MicroBeta2 Plate Counter, PerkinElmer) using a scintillation cocktail (OptiPhaseSuperMix, PerkinElmer).

### LC-MS/MS

The mutant H372^ECL3^C–H89^ECD^C was purified according to our previous study^4^ with the Coomassie blue staining, and summarized below. *Sf*9 membranes were prepared with one wash cycle of hypotonic buffer (25 mM HEPES, pH 7.5, 10 mM MgCl_2_ and 20 mM KCl) in the presence of EDTA-free protease inhibitor cocktail tablets (Roche) and four wash cycles of high-salt buffer with 1 M NaCl supplemented in the hypotonic buffer. Washed membranes were resuspended in 25 mM HEPES, pH 7.5, 10 mM MgCl_2_, 20 mM KCl and 30% glycerol, and incubated with 270 mM of compound NNC0640 and 2 mg ml^−1^ iodoacetamide for 30 min at room temperature. The receptor was solubilized with 1/0.1% (w/v) of DDM/CHS for 2 h at 4 °C. The supernatant was isolated by ultracentrifugation at 504,300 *g* for 30 min at 4 °C, supplemented with 500 mM NaCl and with DDM/CHS adjusted to 0.5/0.05%, and incubated with talon superflow resin overnight at 4 °C in the presence of 5 mM imidazole, pH 7.5. The talon resin was washed with 10 bed volumes of wash buffer 1, and 20 bed volumes of wash buffer 2. The protein was eluted with 2 bed volumes of elution buffer. The band of GCGR proteins was cut into ∼1 mm^3^ slices and put into EP tubes. The gel slices were distained using 50 mM Tris, 4 mM *N*-Ethylmaleimide and 30% acetonitrile (pH 6.5), and dried using Speed-Vac. The dehydrated gel slices were rehydrated with 50 mM Tris, 4 mM NEM (pH 6.5) containing trypsin and chymotrypsin at 20 ng μl^−1^ each for overnight digestion. The reverse phase high-performance liquid chromatography (RP-HPLC) separation was achieved on the Easy NanoLC system (Thermo Fisher Scientific) using a self-packed column (75 μm × 120 mm; 3 μm ReproSil-Pur C18 beads, 120 Å, Dr Maisch GmbH, Ammerbuch, Germany) at a flow rate of 300 nl min^−1^. The mobile phase A of RP-HPLC was 0.1% formic acid in water, and B was 0.1% formic acid in acetonitrile. The peptides were eluted using a 2-h 2–85% B gradient into a nano-ESI LTQ Velos Pro-Orbitrap Elite mass spectrometer (Thermo Fisher Scientific). The mass spectrometer was operated in a data-dependent mode with each full MS scan followed by MS/MS for the 15 most intense ions with the parameters: ⩾+2 precursor ion charge, 2 Da precursor ion isolation window and 35 normalized collision energy of HCD. The following Dynamic Exclusion settings were also used: repeat counts, 1; repeat duration, 120 s; exclusion duration, 180 s. The full mass was scanned in the Orbitrap analyser with *R*=60,000 (defined at *m*/*z* 400), and the subsequent MS/MS analyses were performed in the HCD mode with *R*=15,000; automatic gain control targets were 1 × 10^6^ for Fourier transform mass spectrometry full scan; minimal signal threshold for MS2=5,000. The raw data of in-gel-digested samples were preprocessed using pXtract (http://www.pfindstudio.com/software/pXtract/index.html). The protein database consisted of the H89C/H372C mutant GCGR protein sequence that was used for database searching using the pLink software^67^. The parameters for pLink search were as follows: three missed cleavage sites for trypsin/chymotrypsin per chain; peptide length 4–100 aa; cross-linker disulfide −2.01565 Da on cysteine. pLink search results were filtered by requiring ≤10 p.p.m. deviation in the observed precursor mass from the monoisotopic or the first, second, third or fourth isotopic mass of the matched candidate. Candidate disulfide-linked peptides were filtered with an *E*-value cutoff of 0.01; the interpeptide disulfide bonds were manually checked with following filtering criteria: the two chains contain at least four continuous b or y series ions; and major peaks were assigned to expected ions.

### Simulation systems

The previously reported model of full-length glucagon-bound GCGR^4^ was used as the starting structure of the glucagon-GCGR MD simulations. We extracted the 1.5-μs snapshot from the simulation trajectory of complex system and used the structure of GCGR in this snapshot as the starting structure of the apo-GCGR MD simulation. Then the apo-GCGR structure with mutations Q374R, S379F and A380I was used as the starting structure of the simulation on the ECL3 chimeric apo-GCGR. The apo- and glucagon-bound wild-type GCGR structures and the apo ECL3 chimera GCGR structure were embedded separately in a 100 Å × 100 Å palmitoyloleoylphosphatidylcholine bilayer by aligning the protein's axis of symmetry with the bilayer normal. In each system, lipids located within 1 Å of the proteins were removed. Each system was solvated by TIP3P waters with 0.15 M NaCl. The wild-type apo-GCGR, chimera apo-GCGR and glucagon-GCGR systems include 110,421, 109,950 and 109,972 atoms, respectively.

### MD simulation

MD simulations were performed using the GROMACS4.6.1 package^68^ with isothermal–isobaric (NPT) ensemble and periodic boundary condition. The CHARMM36-CAMP force field^69^ was applied. Energy minimizations were first performed to relieve unfavourable contacts, followed with equilibration steps of 50 ns in total to equilibrate the lipid bilayer and the solvent with restraints to the main chain of the protein and the peptide ligand. The temperature of each system was maintained at 300 K using the v-rescale method with a coupling time of 0.1 ps. The pressure was kept at 1 bar using the Berendsen barostat with *τ*_p_=1.0 ps and a compressibility of 4.5 × 10^−5 ^bar^−1^. SETTLE constraints and LINCS constraints were applied on the hydrogen-involved covalent bonds in water molecules and in other molecules, respectively, and the time step was set to 2 fs. Electrostatic interactions were calculated with the Particle-Mesh Ewald algorithm with a real-space cutoff of 1.4 nm. For each system, one 2-μs production run was performed.

### Normal mode analysis

NMA was conducted using the ElNemo (http://www.igs.cnrs-mrs.fr/elnemo/index.html)^70^, a web interface to the elastic network model-based NMA.

### Analysis of amide proton accessibility

Amide proton accessibility was analysed using the *g_sas* programme and the *g_hbond* programme in the GROMACS4.6.1 package^68^ following the same approach as described previously^40^. An amide proton of GCGR was considered inaccessible if it (1) was involved in a hydrogen bond within the protein structure, (2) was inaccessible (buried) on the protein surface or (3) was accessible only to the lipid bilayer interface. For each MD simulation snapshot extracted at a 100-ps interval from the last 500 ns of the different MD trajectories: (i) hydrogen bonds formed by every main-chain NH group with any other protein atoms were assessed with the *g_hbond* programme, using a hydrogen-bond distance cutoff of 0.35 nm and maximal hydrogen-bond angle deviation of 30°), (ii) solvent accessible surface area of every amide proton was calculated with the *g_sas* programme, using a default water radius of 1.4 Å.

## Additional information

**Accession codes.** Coordinates and structure factors for two electron microscopy structures have been deposited in the EMDataBank under accession codes 6357 (Group 1 as shown in Fig. 1 and Supplementary Fig. 1c) and 6358 (Group 2 as shown in Supplementary Fig. 1d).

**How to cite this article:** Yang, L. *et al*. Conformational states of the full-length glucagon receptor. *Nat. Commun.* 6:7859 doi: 10.1038/ncomms8859 (2015).

## Supplementary Material

Supplementary Figures and Tables Supplementary Figures 1-10 and Supplementary Tables 1-2

Supplementary Movie 1 2 Molecular Dynamics (MD) simulations of apo-GCGR

Supplementary Movie 2 2 Molecular Dynamics (MD) simulations of glucagon (green) bound GCGR (blue)

## Figures and Tables

**Table 1:** Average percentage deuterium uptake of small NNC2648 antagonist and des-His^1^-[Nle^9^-Ala^11^-Ala^16^]-glucagon-NH_2_ peptide antagonist-bound GCGR at 10 s.

**Region**	**Peptide**	**HDX at 10 s (%)**		% Accessible amide protons*	
		**NNC2648 bound**	**des-His**^**1**^**-[Nle**^**9**^**-Ala**^**11**^**-Ala**^**16**^**]-glucagon-NH**_**2**_ **bound**	**apo**	**Glucagon bound**
ECD	FLFEKWKL (31–38)	13.3±5.9	3.8±5.4	20.7±9.5	0.3±1.9
Stalk	IEVQKEVAKM (128–137)	59.6±14.6	6.5±6.2	12.7±6.0	0.9±3.3
ECL1	LRTRYSQKIGDDL (198–210)	45.8±3.3	27.2±2.2	42.6±12.7	28.4±13.7
ICL2	ATLPERSF (256–263)	67.9±8.9	56.3±3.3	51.6±16.9	52.2±10.2
TM6	AKSTLTL (348–354)	2.7±0.3	3.2±2.9	0±0	0±0
ECL3	FVTDEHAQGTLRSAKL (367–382)	44.6±4.2	36.3±2.5	42.3±9.8	35.4±8.1

ECD, extracellular domain; ECL1, first extracellular loop; ECL3, third extracellular loop; GCGR, glucagon receptor; HDX, hydrogen/deuterium exchange; ICL2, intracellular loop 2; MD, molecular dynamics; TM6, transmembrane helix 6.

Predicted average amide proton accessibilities of apo-GCGR and glucagon-GCGR derived from MD simulations.

^*^Values are presented as mean±s.d. of 5,000 snapshots in the last 500-ns simulations of the apo and complex systems, respectively.
