# Supplementary material for: Conformational states of the full-length glucagon receptor
Source: Nat Commun. 2015 Jul 31;6:7859. doi: 10.1038/ncomms8859 (PMC4532856; doi:10.1038/ncomms8859)
Supplement: Supplementary Figures and Tables — Supplementary Figures 1-10 and Supplementary Tables 1-2 [file ncomms8859-s1.pdf]

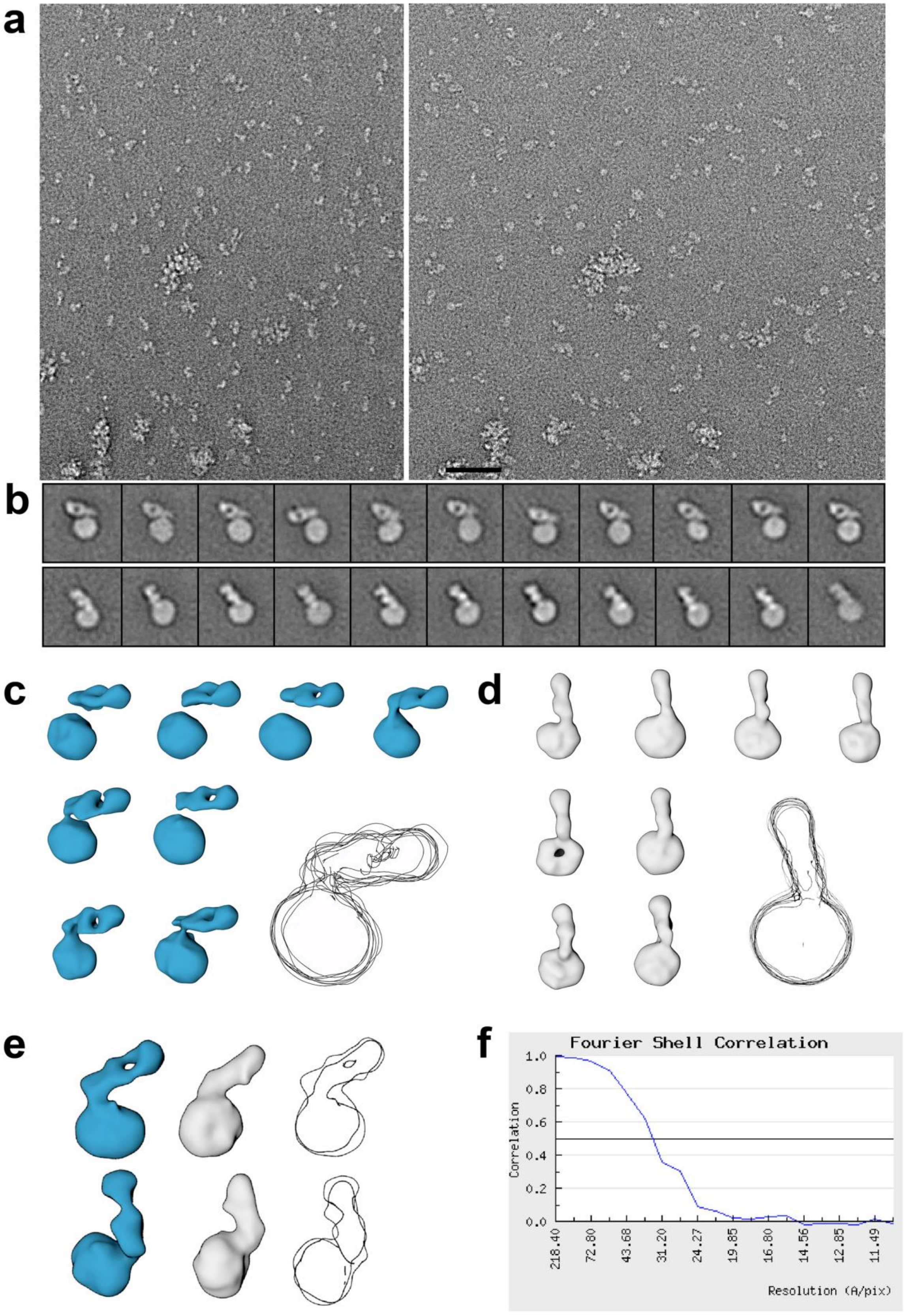

a
b
c
d
e
f
Fourier Shell Correlation
Correlation
Resolution (A/pix)
1.0
0.8
0.6
0.4
0.2
0.0
218.40
72.80
43.68
31.20
24.27
19.85
16.80
14.56
12.85
11.49

**Supplementary Figure 1 (previous page). EM analysis of full-length GCGR.** (**a**) Exemplary tilt pair images of the GCGR–mAb23 complex acquired for Random Conical Tilt (RCT) reconstruction (left: -50°,right: 0°); a total of 648 tilt pairs were collected at 62,000× magnification corresponding to 0.273 nm pixel$^{-1}$. Scale bar is 50 nm. (**b**) Exemplary 2D class averages from untilted micrographs. Two principal orientations of the mAb23 Fab are identified (mAb23 is rotated by ~ 90° between the top row (Group 1) and bottom row (Group 2) of class averages). (**c**) A selection of 3D density maps generated from the Group 1 class averages using RCT reconstructions (see Figure 1 for comparison); superposition of the 3D envelopes (grey overlay) indicates the similarity of the shape of the GCGR-mAb23 complex in all maps. (**d**) RCT reconstructions from Group 2 class averages and map superposition. (**e**) Comparison between the maps calculated from the two different preferred orientation shows good agreement and indicates that mAb23 is bound to GCGR in a stable conformation. (**f**) Fourier Shell Correlation plot used to calculate the resolution of the 3D map shown in Figure 1.

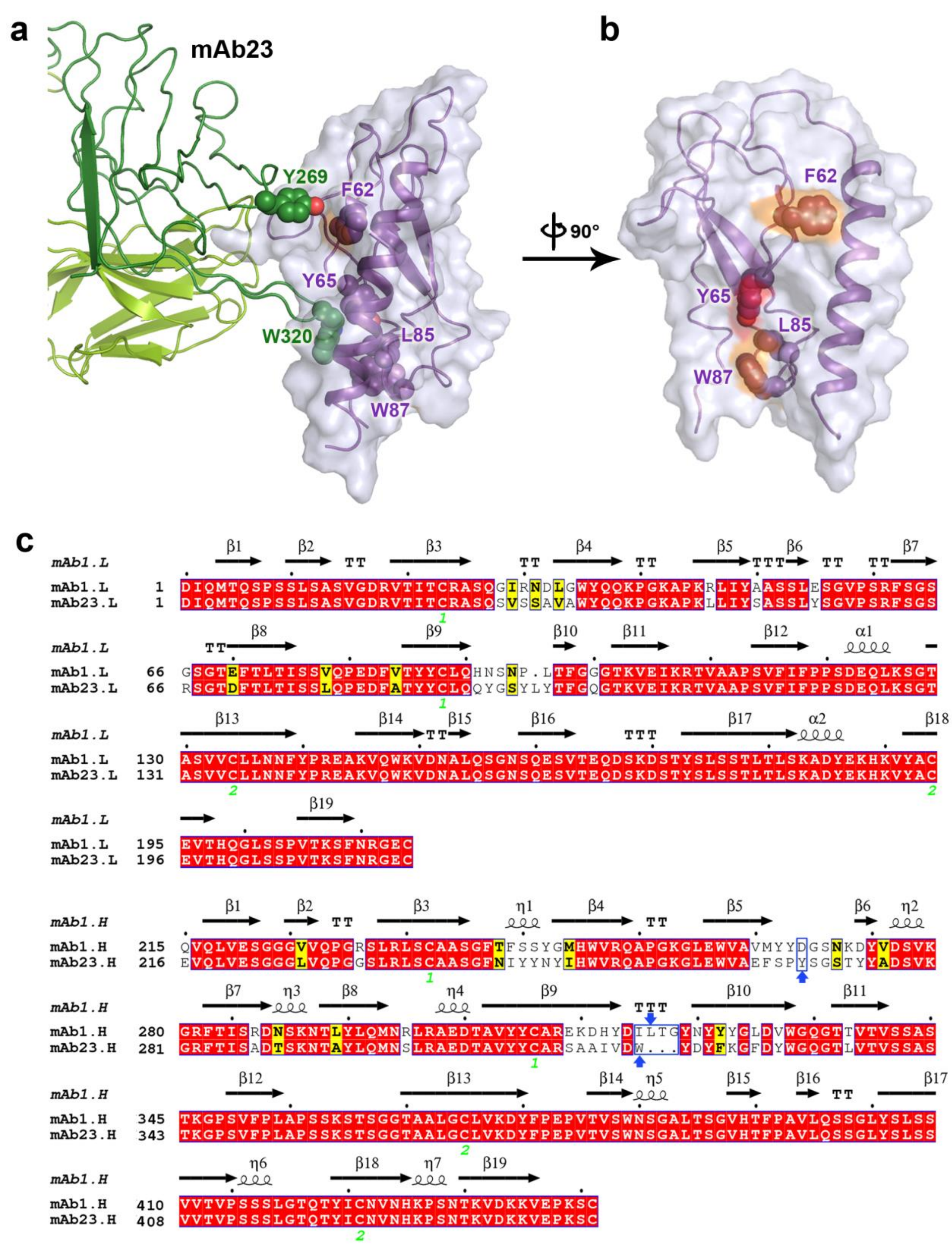


**Supplementary Figure 2. The mAb23-ECD bound model and sequence alignments of mAb1 and mAb23.** (**a,b**) The mAb23-ECD interactions in the model. Y65, L85, and W87 of the ECD are in the vicinity of the H3 loops of mAb23. The heavy chain and the light chain of the mAb23 Fab are colored in green and light green respectively, while the ECD is in purple cartoon with a transparent white surface. Important residues in the interface are depicted in spheres and their surfaces are colored red (Y65) or orange (F62,

L85, W87). (**c**) Sequence alignments of the heavy chain and the light chain of mAb1 and mAb23 with secondary structure elements for mAb1 indicated on top (PDB:4ERS). Y269 and W320 in mAb23 as well as L320 in mAb1 are marked by blue arrows. Identical residues are shown as white letters on red background. Partially conserved residues are shown as black letters on yellow background. Cysteine pairs forming disulfide bonds are indicated by green numbers at the bottom.

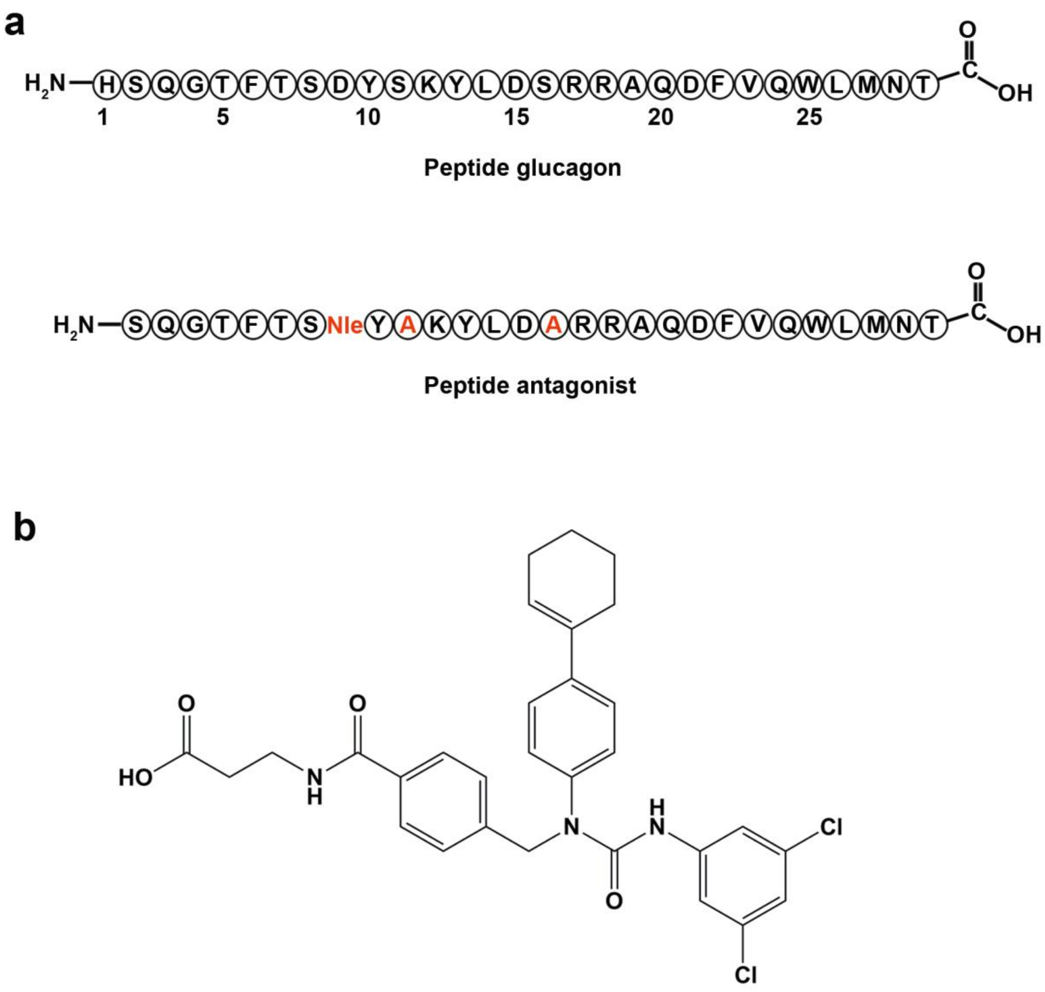


**Supplementary Figure 3. Peptide antagonist and the small molecule antagonist (NNC2648). (a)** The amino acid sequences of glucagon peptide and the peptide antagonist (desHis(1)-Nle(9)-Ala(11,16)-glucagon) used in the HDX studies. (**b**) The molecular structure of GCGR antagonist NNC2648.

## HDX percent values for full length GCGR bound to NNC2648 in LMNG

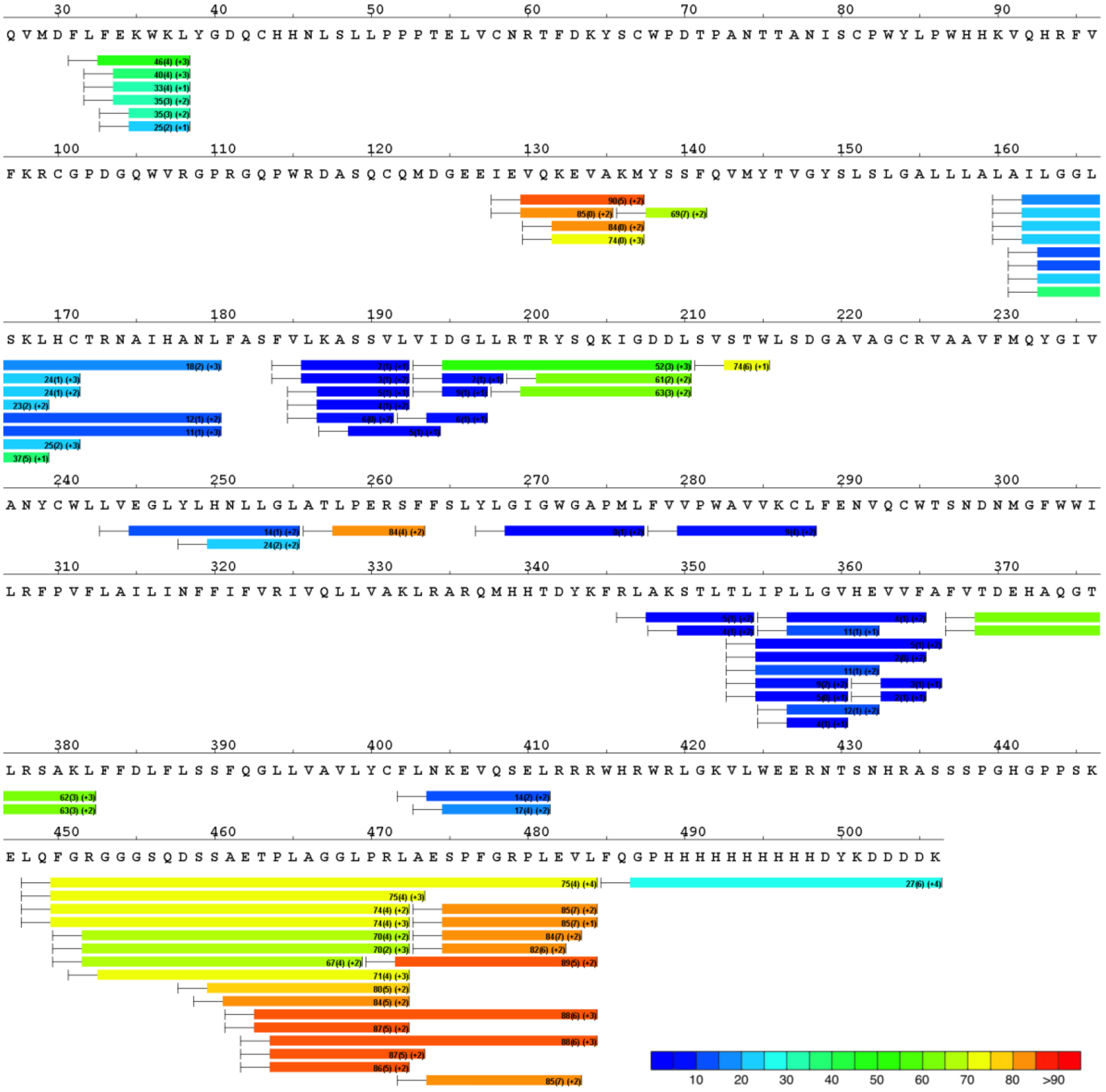


**Supplementary Figure 4. Average percent deuterium values in HDX studies with wild-type GCGR.** (**a**) GCGR peptide sequences are color coded according to the average HDX percent values for full length GCGR bound to NNC2648 in LMNG.

**Average percent change in HDX values of full length GCGR bound to NNC2648 vs. full length GCGR bound to (desHis(1)-Nle(9)-Ala(11,16)-glucagon)**

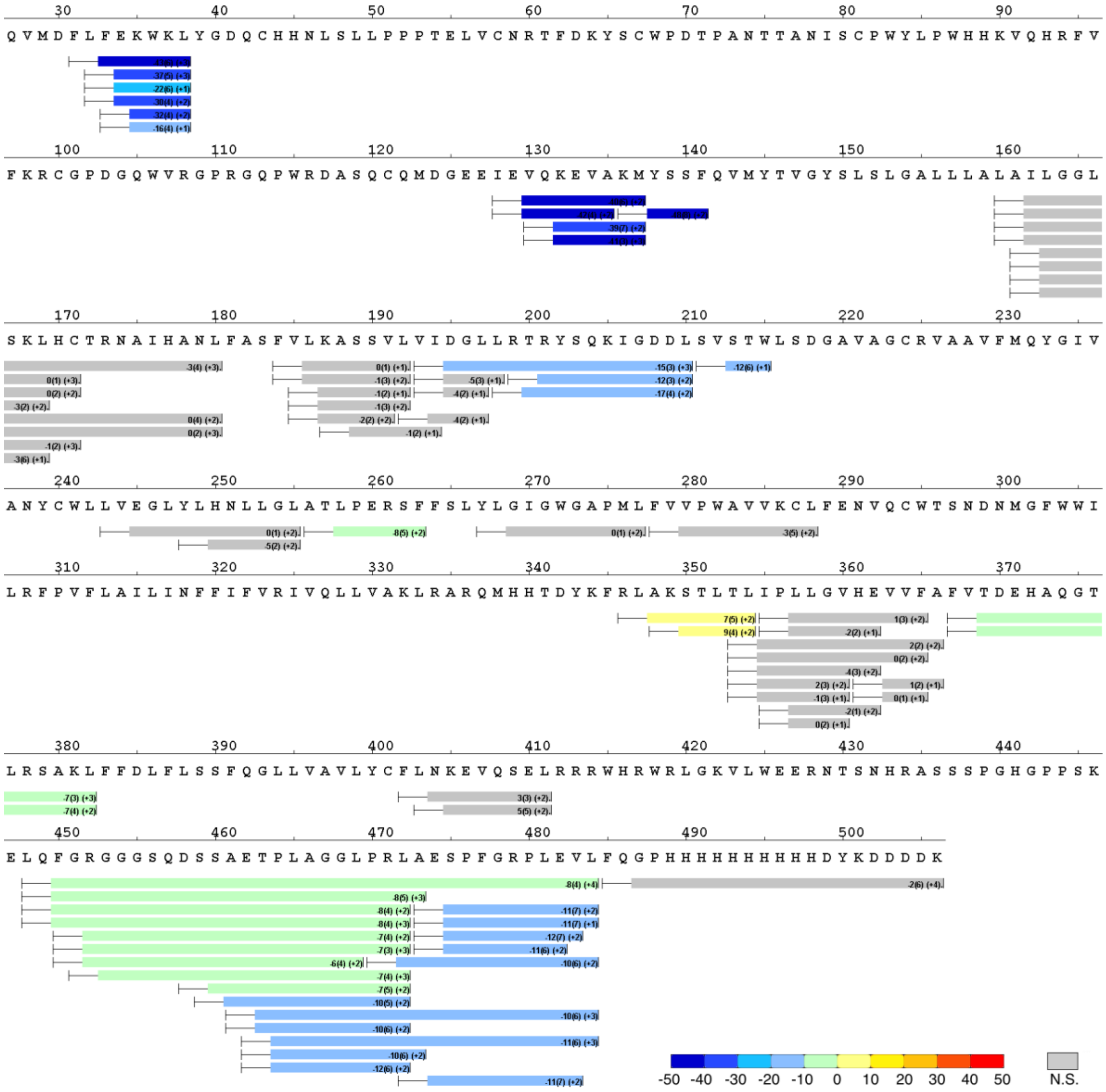

**Supplementary Figure 4 (continued). Average percent deuterium values in HDX studies with wild-type GCGR.** (**b**) GCGR peptide sequences are color coded according to the average percent change in HDX values for full length GCGR bound to NNC2648 vs full length GCGR bound to (desHis(1)-Nle(9)-Ala(11,16)-glucagon) peptide antagonist.

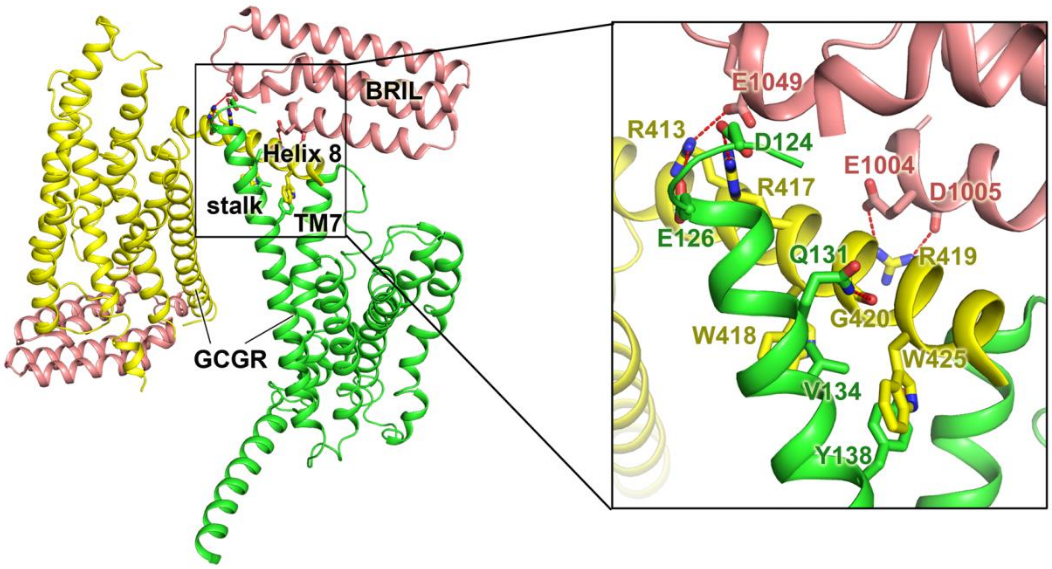


**Supplementary Figure 5. The conformation of the TM1 stalk in the GCGR 7TM crystal structure.** The TM1 stalk is stabilized by the BRIL fusion protein and helix 8 of the adjacent symmetric unit in the GCGR 7TM crystal structure (PDB: 4L6R). The 7TMs of the two BRIL-GCGR (ΔECD/ΔC) molecules are colored in yellow and green, respectively, and the BRIL protein is colored in salmon. W245 and W418 in helix 8 of the adjacent GCGR form hydrophobic interactions with Y138 and V134 in the stalk. Meanwhile, G420, R417 and R413 in helix 8 can form hydrogen bonds with Q131, D124 and E126, respectively. In addition, helix 8 forms polar interactions with BRIL (such as R413-E1049, R419-E1004 and R419-D1005), which promote the stabilization of the TM1 stalk.

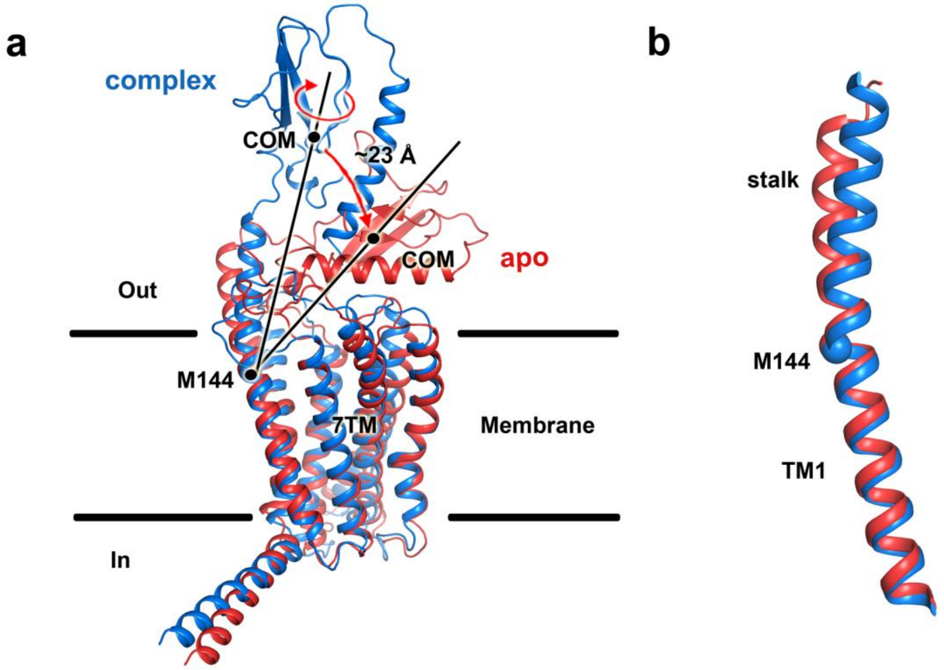


**Supplementary Figure 6. Conformational changes in the simulation on the *apo*-GCGR.** (**a**) Orientation changes of the ECD with respect to the 7TM in the apo system (red) compared with the average structure of the glucagon-GCGR (blue). For clarity, glucagon in the complex system is hidden. The average structures in each system (extracted from the last 500-ns trajectory of each simulation) are superimposed using the main-chain atoms of the 7TM helices. (**b**) Conformation changes of TM1 and the TM1 stalk region (125-136) under the superposition in panel a. C$\alpha$ atoms of the hinge residue M144 in these structures are depicted in spheres.

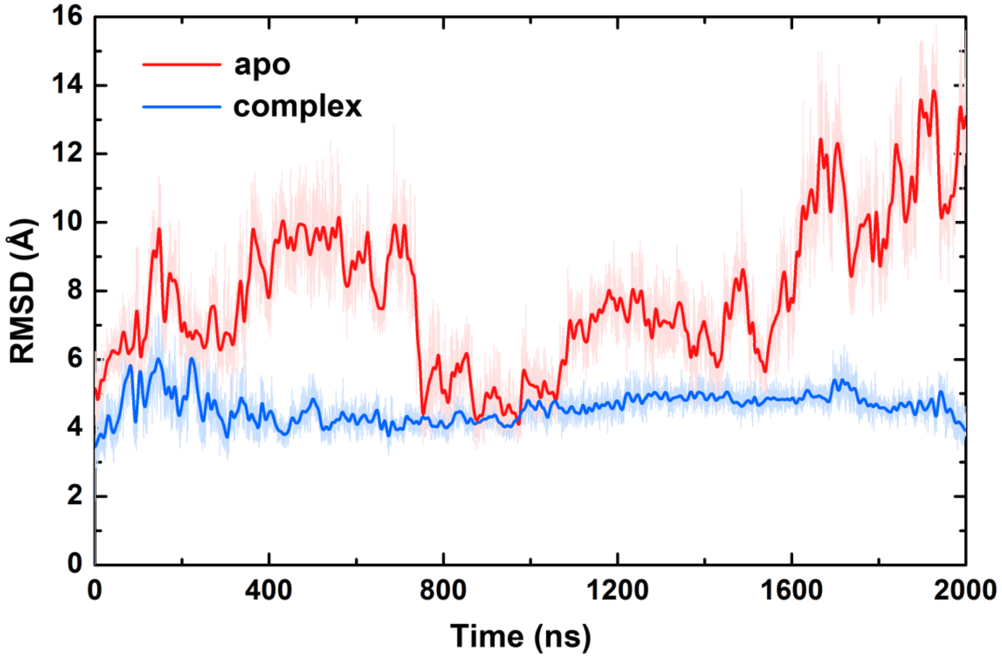


**Supplementary Figure 7. Root mean square deviations (RMSD) of ECL1 (198-218) in the simulations on glucagon-GCGR (blue) and apo-GCGR (red).** Apparently, ECL1 is more stable in the glucagon-GCGR than in the apo-GCGR.

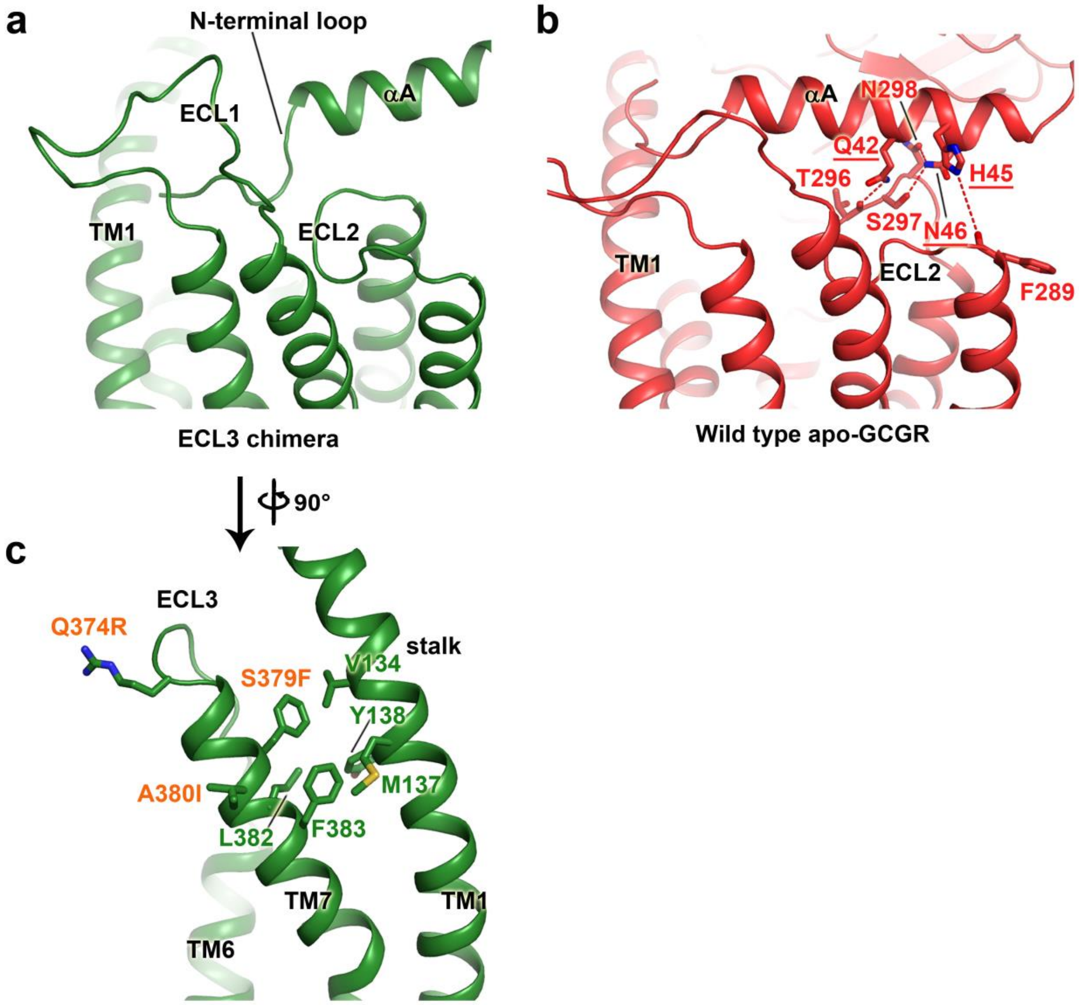


**Supplementary Figure 8. Interactions in the simulation on the ECL3 chimera.** (**a**) There are no stable interactions between the N-terminal loop of the ECD and ECL1 and ECL2 in the closed-like structure of the chimera, as these loops are very dynamic. (**b**) ECL2 mainly contacts with αA of the ECD in the wild-type closed state, and there are relatively stable interactions between them. The snapshot at 300 ns in the simulation on the chimera and the structure of conf2 in Figure 3 are used. For clarity, residues in the ECD are underlined. Residues involved in interactions are shown in sticks. (**c**) Stronger hydrophobic interactions between TM7 and the TM1 stalk region in the chimera. Mutations Q374R, S379F and A380I are labeled in orange. S379F contributes to the stronger hydrophobic patterns between TM7 and the TM1 stalk region, which consists of L382, F383, M137, Y138 and V134 in the wild-type *apo*-GCGR.

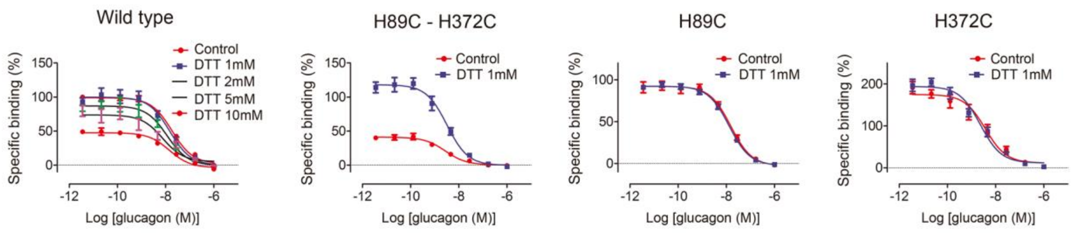


**Supplementary Figure 9. The intervention of glucagon binding by disulfide cross-linking studies.** The left panel represented the binding curves of wild type GCGR with DTT in different concentrations. Then the binding affinity of H89C/H372C, H89C and H372C mutant GCGR were determined in the presence or absence of 1 mM DTT. Data are expressed as a percentage of specific $^{125}$I-glucagon binding with wild type GCGR in the presence of 3.57 pM unlabeled peptide. Each point represents the mean value ± SEM of at least three independent experiments done in duplicate.

**a** GCGR-(H89C-H372C) (protein sequence)
*MPPCQPQRPLLLLLLLLACQPQVPSA*QVMDFLFEKWKLYGDQCHHNLSLLPPPTELVCNRTFDKYSCWPDTP
ANTTANISCPW**<u>YLPWHCK</u>**VQHRFVFKRCGPDGQWVRGPRGQPWRDASQCQMDGEEIEVQKEVAKMYSS
FQVMYTVGYSLSLGALLLALAILGGLSKLHCTRNAIHANLFASFVLKASSVLVIDGLLRTRYSQKIGDDLSVS
TWLSDGAVAGCRVAAVFMQYGIVANYCWLLVEGLYLHNLLGLATLPERSFFSLYLGIGWGAPMLFVVPWAV
VKCLFENVQCWTSNDNMGFWWILRFPVFLAILINFFIFVRIVQLLVAKLRARQMHHTDYKFRLAKSTLTLIP
LLGVHEVVF**<u>AFVTDECAQGTLR</u>**SAKLFFDLFLSSFQGLLVAVLYCFLNKEVQSELRRRWHRWRLGKVLWE
ERNTSNHRASSSPGHGPPSKELQFGRGGGSQDSSAETPLAGGLPRLAESPF

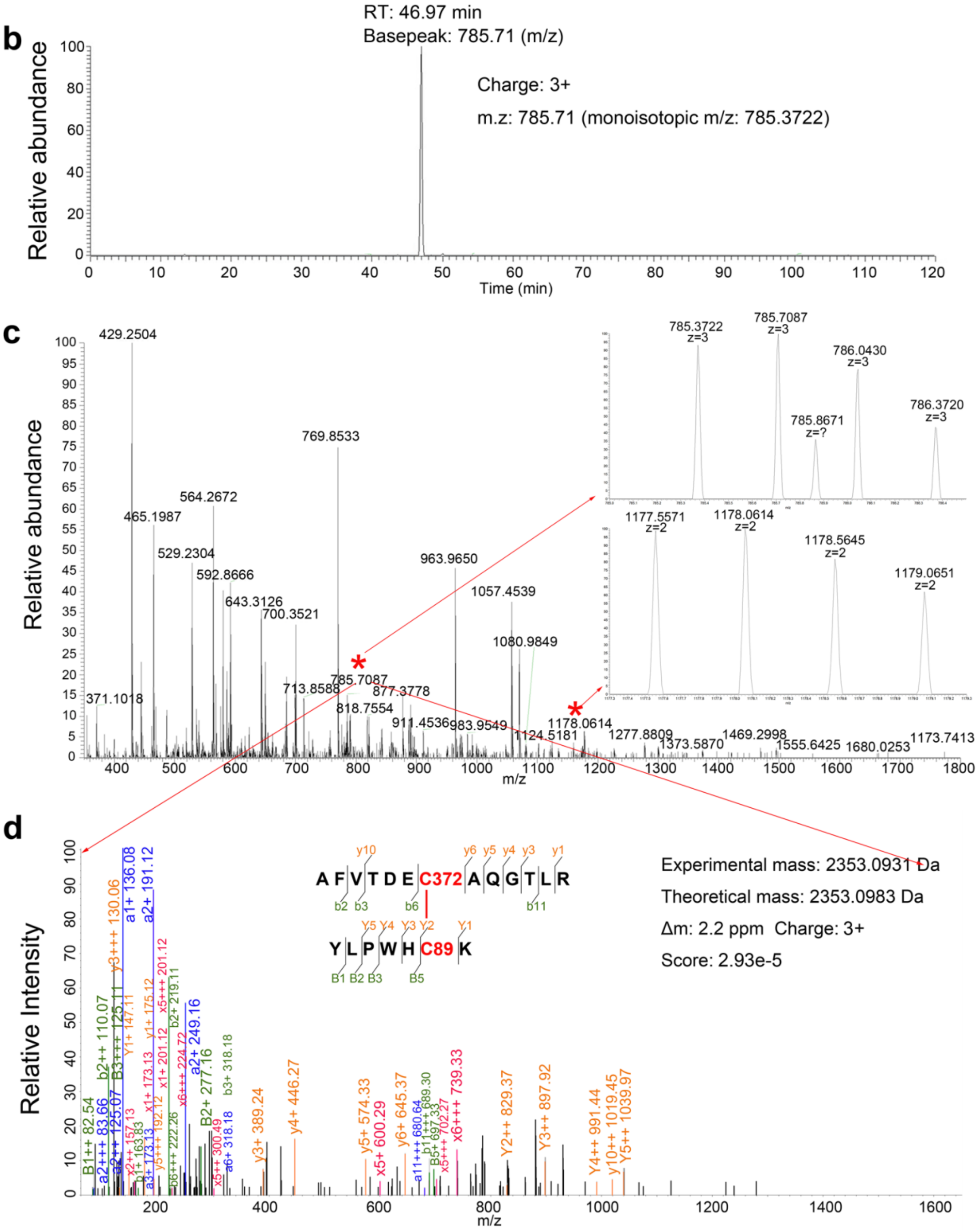

**Supplementary Figure 10 (previous page). Mass spectrometry characterization of C89-C372 disulfide bond formation in H89C/H372C GCGR mutant. (a)** The schematic graph of the C89-C372 disulfide bond in H89C/H372C GCGR mutant; **(b)** Nano-liquid chromatography/electrospray ionization LTQ Velos Pro-Orbitrap Elite mass spectrometric analysis extracted ion chromatogram of GCGR from *Sf*9 cells with chymotrypsin and trypsin digestion, representing the triply charged cross-linked peptide between YLPWHC(89)K and AFVTDEC(372)AQGTLR through a disulfide bond. **(c)** Mass spectrum of nano-LC-MS at the retention time of 46.83 min. The triply charged (*m*/*z* 785.0-786.5, Theoretical mass: 2,353.0983 Da, Experimental mass: 2,353.0931) and doubly charged (*m*/*z* 1177.3-1179.3, Theoretical mass: 2,353.0983 Da, Experimental mass: 2,353.0986) peaks, which are corresponding to the target disulfide-containing peptide. **(d)** MS/MS spectra of the HCD fragmentation of the triply charged disulfide-containing peptide are shown. *b*, *y*, B and Y are types of fragment ions, top-left panel: graphical fragment map that correlates the fragmentation ions to the peptide sequence. The disulfide-linked cysteines C89 (ECD) and C372 (ECL3) are shown in *red*.

**Supplementary Table 1: Binding data of GCGR mutants with or without DTT treatment.**

| GCGR construct | Span[a] (Wild type%, Mean±SEM) | | $IC_{50}$ (nM, Mean±SEM) | |
|---|---|---|---|---|
| | Control | 1mM DTT | Control | 1mM DTT |
| **Wild type** | 100 | 97.91±3.07 | 15.07±2.72 | 9.48±1.71 |
| **H89C/H372C** | 43.74±5.41 | 127.06±15.77 | 2.76±0.27 | 3.16±0.51 |
| **H89C** | 101.61±9.74 | 101.09±3.8 | 15.34±1.53 | 13.12±1.15 |
| **H372C** | 163.23±23.6 | 186.62±10.93 | 4.22±2.14 | 2.76±1.17 |

[a]Specific $^{125}$I-glucagon binding (span) is defined as the window between the highest (3.57 pM unlabeled ligand) and lowest binding (1 μM unlabeled ligand). The values shown are means ± SEM of at least three independent experiments done in triplicate.

**Supplementary Table 2: Differences in average % deuterium values in HDX studies of apo H89C/H372C mutant vs. GCGR (desHis(1)-Nle(9)-Ala(11,16)-glucagon) peptide antagonist bound wild-type GCGR .**

| Region | Peptide residue (charge) | ΔHDX (H89C/H372C GCGR - WT GCGR + Glucagon peptide) |
|---|---|---|
| ECD | 32-38 (+3) | -39 ± 5 |
| ECD | 33-38 (+2) | -37 ± 4 |
| TM1 Stalk | 128-137 (+2) | -34 ± 6 |
| ICL1 | 160-169 (+2) | -15 ± 3 |
| ECL1 | 193-210 (+3) | -19 ± 4 |
| ECL1 | 198-210 (+2) | -21 ± 4 |
| ICL2 | 248-255 (+2) | -22 ± 3 |
| C-term | 448-469 (+3) | 3 ± 4 |
| C-term | 448-472 (+2) | -10 ± 5 |
| C-term | 462-472 (+2) | -7 ± 4 |
| C-term | 472-483 (+2) | -10 ± 5 |
| C-term | 473-482 (+2) | -11 ± 5 |
| C-term | 473-483 (+2) | -20 ± 6 |
| C-term | 473-484 (+2) | -10 ± 6 |
