# Supplementary figures and images for: Conformational states of the full-length glucagon receptor
Source: Nat Commun. 2015 Jul 31;6:7859. doi: 10.1038/ncomms8859 (PMC4532856; doi:10.1038/ncomms8859)

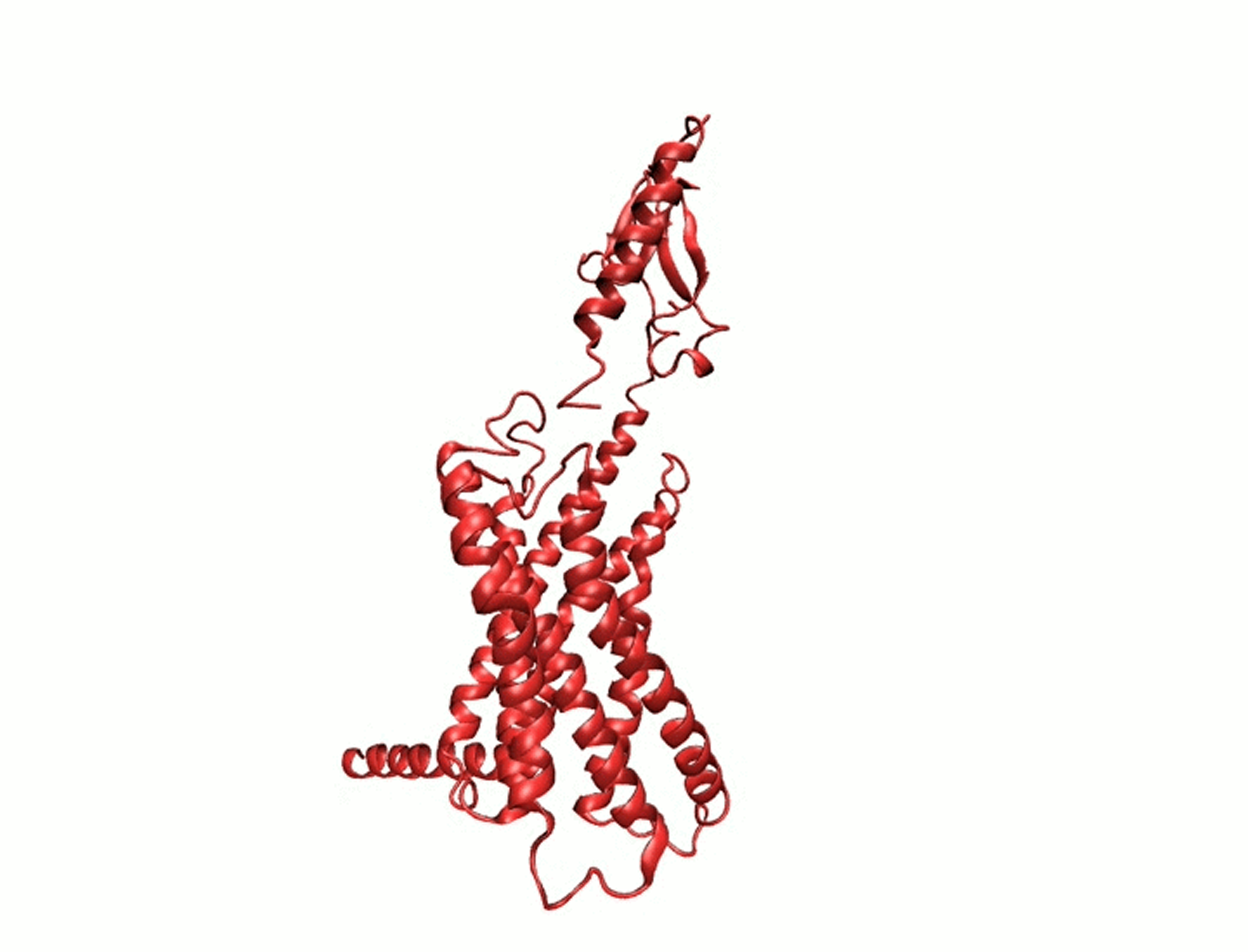

Supplement: Supplementary Movie 1 — 2 Molecular Dynamics (MD) simulations of apo-GCGR [file ncomms8859-s2.tif]

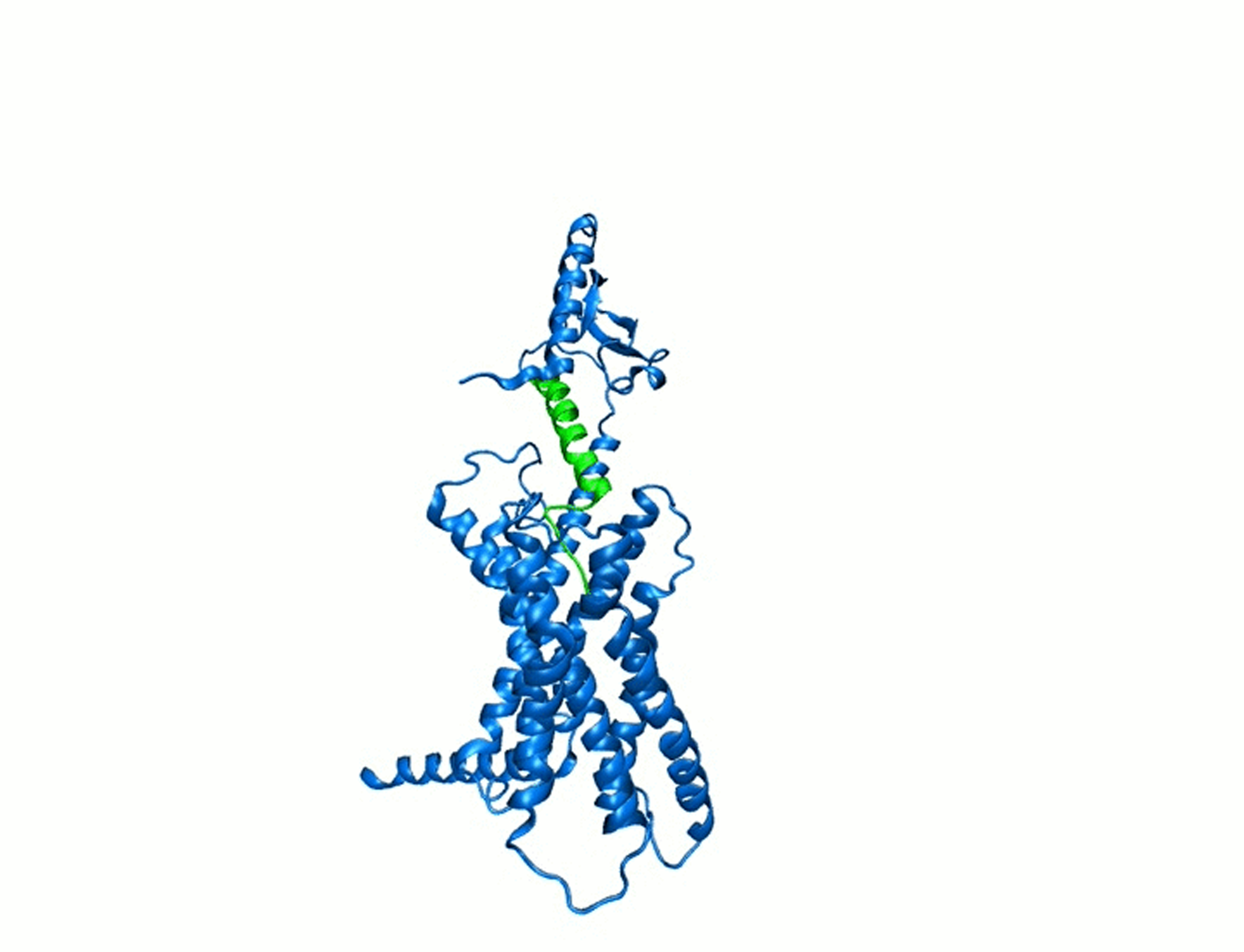

Supplement: Supplementary Movie 2 — 2 Molecular Dynamics (MD) simulations of glucagon (green) bound GCGR (blue) [file ncomms8859-s3.tif]
